# Ecology of community reassembly: Movements and diets of megafauna during a decade of restoration in Mozambique

**DOI:** 10.1002/ecy.70386

**Published:** 2026-04-12

**Authors:** Matthew C. Hutchinson, Reena H. Walker, Joel O. Abraham, Justine A. Becker, Dominique D. Gonçalves, Ciara M. Nutter, Johan Pansu, Erin M. Phillips, Arjun B. Potter, Beto Soares Tenente, Marc E. Stalmans, Ryan A. Long, Robert M. Pringle

**Affiliations:** ^1^ Department of Ecology & Evolutionary Biology Princeton University Princeton New Jersey USA; ^2^ Department of Life & Environmental Sciences University of California Merced California USA; ^3^ Department of Fish & Wildlife Sciences University of Idaho Moscow Idaho USA; ^4^ Department of Ecology & Institute on Ecosystems Montana State University Bozeman Montana USA; ^5^ Durrell Institute of Conservation and Ecology, University of Kent Kent UK; ^6^ Department of Scientific Services Gorongosa National Park Sofala Mozambique; ^7^ Université Claude Bernard Lyon 1, CNRS, ENTPE, UMR 5023 LEHNA Villeurbanne France

**Keywords:** African savannas, animal movement ecology, ecosystem restoration, fecal eDNA metabarcoding diet analysis, habitat selection, herbivore foraging behavior, plant–animal interaction networks, resource selection function, species interactions, trophic rewilding, ungulate space use, wildlife management

## Abstract

The last decade has brought a worldwide surge of interest in rewilding—the repopulation of large herbivores and carnivores—as a strategy for conserving species and reviving ecosystem functions. Rewilding initiatives, if closely monitored, can provide unique insights into the ecology of the world's largest animals at otherwise impossible spatial and temporal scales. Capitalizing on these opportunities, and developing a knowledge base to guide future restoration efforts, requires the collection and dissemination of long‐term data that document community reassembly. To date, such data are virtually nonexistent: most megafaunal restoration projects are nascent and/or have not been rigorously monitored. Since 2008, the Gorongosa Restoration Project, in Mozambique's Gorongosa National Park, has facilitated the recovery of megafauna populations that were severely depleted or extirpated during the country's civil war (1977–1992). For over a decade, we have monitored Gorongosa's large‐herbivore populations to understand how animal behavior and trophic interactions change as communities reassemble. Here, we present spatiotemporally explicit data sets on the movements and diets of large herbivores in Gorongosa between 2013 and 2025, along with annual rainfall data. This period encompassed extremes of climate (including some of the driest and wettest years on record) and the reintroduction, starting in 2018, of locally extinct apex predators and scavengers: African wild dog (*Lycaon pictus*), leopard (*Panthera pardus*), spotted hyena (*Crocuta crocuta*), and side‐striped jackal (*Lupulella adusta*). We used GPS telemetry to monitor 277 herbivores of seven species (listed below with number of individuals collared, median duration of tracking, and median number of locations per individual): Cape bushbuck (*Tragelaphus sylvaticus*: 103 individuals; 280 days; 6646 fixes), nyala (*T. angasii*: 37 individuals; 306 days; 6789 fixes), greater kudu (*T. strepsiceros*: 80 individuals; 300 days; 17,365 fixes), common eland (*T. oryx*: 10 individuals; 334 days; 15,783 fixes), waterbuck (*Kobus ellipsiprymnus*: 22 individuals; 13 days; 2877 fixes), plains zebra (*Equus quagga*: 7 individuals; 212 days; 1171 fixes), and African savanna elephant (*Loxodonta africana*: 18 individuals; 706 days; 33,122 fixes). For 295 individuals that were immobilized during this work, we present morphological measurements (chest girth, body length, hind‐foot length, weight), reproductive status and nutritional condition (ultrasound measurements, palpation scores), and fate (mortality date and cause, if known). For diet analysis, we used DNA metabarcoding to identify and quantify the relative abundances of plant taxa in 3785 fecal samples from 27 mammal species belonging to 11 families and 7 orders. In all, we recorded 516 food‐plant taxa from at least 87 plant families and 39 orders. For Gorongosa's 15 most common large herbivores, the median sampling depth was 216 fecal samples per species (interquartile range 156–279); the overall median sampling depth was 92 samples per species (range 1–499). We include basic metadata collected in the field (e.g., date, time, GPS location, animal sex, and age) along with laboratory notes and information on plant taxonomic identification. These data are valuable not just as a window on one ecosystem's recovery from armed conflict, but also as a resource for macroecology, meta‐analysis, and synthetic studies of animal movement, diet, and the dynamics of community reassembly. The data are freely available for use and this paper should be cited whenever data are reused; see Data S1: Metadata S1: Class III.B.4 for additional details.

## AUTHOR CONTRIBUTIONS

Conceptualization: RMP, RAL. Investigation: MCH, RHW, JOA, JAB, DDG, CMN, JP, EMP, ABP, BST, MES, RAL, RMP. Data curation: MCH, RHW. Project administration and supervision: RMP, RAL. Writing ‐ original draft: MCH, RHW, RAL, RMP. Writing ‐ review and editing: MCH, RHW, JOA, JAB, DDG, CMN, JP, EMP, ABP, BST, MES, RAL, RMP. All authors reviewed and approved the final submission.

## CONFLICT OF INTEREST STATEMENT

The authors declare no conflicts of interest.

## Supporting information


Data S1.

## Data Availability

Filtered data, metadata, and data‐cleaning scripts are provided as Supporting Information in Data S1 and in Dryad at https://doi.org/10.5061/dryad.m905qfv85. Movement data are also deposited on Movebank (Study ID#: 7359354918). Raw sequencing data from DNA metabarcoding (datasets 18 and 19 in Metadata S1) are omitted from Data S1 due to size constraints and are instead provided only in the Dryad repository.

