# Supplementary material for: Ecology of community reassembly: Movements and diets of megafauna during a decade of restoration in Mozambique
Source: Ecology. 2026 Apr 12;107(4):e70386. doi: 10.1002/ecy.70386 (PMC13071350; doi:10.1002/ecy.70386)
Supplement: Supplementary file 1 — Data S1. [file ECY-107-e70386-s001.zip › Metadata_S1.pdf]

Data Paper published in *Ecology*, an Ecological Society of America journal

## Metadata S1

# Ecology of community reassembly: Movements and diets of megafauna during a decade of restoration in Mozambique

Matthew C. Hutchinson, Reena H. Walker, Joel O. Abraham, Justine A. Becker, Dominique D. Gonçalves, Ciara M. Nutter, Johan Pansu, Erin M. Phillips, Arjun B. Potter, Beto Soares Tenente, Marc E. Stalmans, Ryan A. Long, Robert M. Pringle

**Class I. Data Set Descriptors**

**A. Data set identity:** Ecology of community reassembly: Movements and diets of megafauna during a decade of restoration in Mozambique

**B. Data set identification code:** Dryad, https://doi.org/10.5061/dryad.m905qfv85

**C. Data set description**

**1. Originators:**

1. Robert M. Pringle, Department of Ecology and Evolutionary Biology, Princeton University, Princeton, NJ 08544, United States of America. Email: rpringle@princeton.edu
2. Ryan A. Long, Department of Fish and Wildlife Sciences, University of Idaho, Moscow, ID 83844, United States of America. Email: ralong@uidaho.edu

**2. Abstract:** The last decade has brought a worldwide surge of interest in rewilding—repopulation of large herbivores and carnivores—as a strategy for conserving species and reviving ecosystem functions. Rewilding initiatives, if closely monitored, can provide unique insights into the ecology of the world's largest animals at otherwise impossible spatial and temporal scales. Capitalizing on these opportunities, and developing a knowledge base to guide future restoration efforts, requires the collection and dissemination of long-term data that document community reassembly. To date, such data are virtually nonexistent: most megafaunal restoration projects are nascent and/or have not been rigorously monitored. Since 2008, the Gorongosa Restoration Project, in Mozambique's Gorongosa National Park, has facilitated the recovery of megafauna populations that were severely depleted or extirpated during the country's civil war (1977–1992). For over a decade, we have monitored Gorongosa's large-herbivore populations to understand how animal behavior and trophic interactions change as communities reassemble. Here, we present spatiotemporally explicit data sets on the movements and diets of large herbivores in Gorongosa between 2013 and 2025, along with annual rainfall data. This period encompassed extremes of climate (including some of the driest and wettest years on record) and the reintroduction, starting in 2018, of locally extinct apex predators and scavengers: African wild dog (*Lycaon pictus*), leopard (*Panthera pardus*), spotted hyena (*Crocuta crocuta*), and side-striped jackal (*Lupulella adusta*). We used GPS telemetry to monitor 277 herbivores of seven species (listed below with number of individuals

collared, median duration of tracking, and median number of locations per individual): Cape bushbuck (*Tragelaphus sylvaticus*: 103 individuals; 280 days; 6,646 fixes), nyala (*T. angasii*: 37 individuals; 306 days; 6,789 fixes), greater kudu (*T. strepsiceros*: 80 individuals; 300 days; 17,365 fixes), common eland (*T. oryx*: 10 individuals; 334 days; 15,783 fixes), waterbuck (*Kobus ellipsiprymnus*: 22 individuals; 13 days; 2,877 fixes), plains zebra (*Equus quagga crawshayi*: 7 individuals; 212 days; 1,171 fixes), and African savanna elephant (*Loxodonta africana*: 18 individuals; 706 days; 33,122 fixes). For 295 individuals that were immobilized during this work, we present morphological measurements (chest girth, body length, hind-foot length, weight), reproductive status and nutritional condition (ultrasound measurements, palpation scores), and fate (mortality date and cause, if known). For diet analysis, we used DNA metabarcoding to identify and quantify the relative abundance of plant taxa in 3,785 fecal samples from 27 mammal species representing 11 families and 7 orders. In all, we recorded 516 food-plant taxa from at least 87 families and 39 orders. For Gorongosa's 15 most common large herbivores, the median sampling depth was 216 samples per species (IQR 156–279); the overall median sampling depth was 92 samples per species (range 1–499). We include basic metadata collected in the field (e.g., date, time, location, animal sex and age) along with laboratory notes and information on plant taxonomic identification. These data are valuable not just as a window on one ecosystem's recovery from armed conflict, but also as a resource for macroecology, meta-analysis, and synthetic studies of animal movement, diet, and the dynamics of community reassembly. The data are freely available for use and this paper should be cited when data are reused; see Data S1: Metadata S1: Class III.B.4 for additional details.

**D. Key words/phrases:** African savannas; animal movement ecology; ecosystem restoration; fecal eDNA metabarcoding diet analysis; habitat selection; herbivore foraging behavior; plant-animal interaction networks; resource selection function; species interactions; trophic rewilding; ungulate space use; wildlife management

## Class II. Research origin descriptors

### A. Overall project description

**1. Identity:** Ecology of community reassembly: Movements and diets of megafauna during a decade of restoration in Mozambique

**2. Originators:** R.M. Pringle and R.A. Long (see *Class I.C.1*)

**3. Period of study:** 2013–2025. Ongoing.

**4. Objectives:** To investigate the movements, foraging, and species interactions of large mammalian herbivores, as well as the spatial and temporal dynamics of diet and habitat selection in response to large-scale ecological restoration.

**5: Abstract:** see *Class I.C.2*.

**6: Sources of funding:** Data presented here are the result of support from the United States National Science Foundation (DEB-1355122, DEB-1501306, IOS-1656527, and DEB-2225088 to RMP; IOS-1656642 to RAL; DGE-1656466 to ABP); the Greg Carr Foundation; the Cameron Schrier Foundation; Princeton University (Innovation Fund for New Ideas in Natural Sciences, the Institute for African Studies, the Institute for International and Regional Studies); the High Meadows Environmental Institute at Princeton University (Grand Challenges Program in Water and in Biodiversity, the Randall and Mary Hack '69 Award for Water and the Environment, the Becky Colvin Memorial Award); the Carbon Mitigation Initiative; the Rufford Foundation; the Campizondo Foundation; Artiopart; Save the Elephants; the Sherwood Family Foundation; the Porter Family Fund for Sustainability Science; the National Geographic Society (9459-14 to Jennifer A. Guyton, EC-412R-18 to MCH, 9291-13 to RMP, WW-070ER-17 to JAB, WW-268C-17 to RAL), and the Gorongosa Project.

**B. Specific subproject description**

**1. Site description:** All data originate from Parque Nacional da Gorongosa (Gorongosa National Park, 'PNG') in central Mozambique.

**a. Site type:** Subhumid tropical savanna, grassland, and miombo woodland.

**b. Geography:** PNG is located in Sofala Province, Mozambique, between latitudes -18.2° and -19.2° and longitudes 34.0° and 35.0°. The roughly quadrangular 3,700-km$^2$ park was established in 1960 and encompasses the southern tip of the Great Rift Valley (10–30 m above sea level, ASL) along with its eastern and western escarpments (up to 400 m ASL). In 2010, a non-contiguous 370-km$^2$ area on Serra da Gorongosa (Mount Gorongosa), an 1,870 m ASL massif 20 km west of PNG's northern boundary, was added to the park. For convenience, we use 'PNG' exclusively to denote the original, lower-elevation portion of the park, where our research was conducted (**Fig. 1**). PNG and Mount Gorongosa are embedded within a 5,400-km$^2$ buffer zone, designated for human development and sustainable use of natural resources (IUCN Category VI), which is inhabited by an estimated 250,000 people.

**c. Habitat:** PNG spans multiple habitat types that are broadly defined by the geology of the Rift Valley, which runs through the park from north to south (**Fig. 1a**). At the center, floodplain grasslands surround Lake Urema from 8–20 m ASL (Stalmans and Beilfuss 2008, Daskin et al. 2016, Lötter et al. 2023, Walker et al. 2023a, 2023b). These are sparsely wooded or treeless plains and mudflats that flood annually for extended periods. With increasing distance from the lake, semiaquatic and hygrophilous forbs give way to lawn and bunch grasses. From around 20 m ASL (Walker et al. 2023a, 2023b), where inundation is still frequent but shallower and more transient, floodplains transition into a mix of palm thickets (*Hyphaene petersiana*) and open fine-leaved savanna dominated by acacias (*sensu lato*, notably *Vachellia xanthophloea* and *Faidherbia albida*). The floodplain and transitional savanna habitats together account for roughly 650 km$^2$ (**Fig. 1a**). Upwards of 25 m ASL, open savannas blend into mixed savanna woodlands on escarpment-derived alluvial and colluvial deposits, which collectively occupy around 1,670 km$^2$ (Lötter et al. 2023; **Fig. 1a**). These Rift Valley woodlands feature both fine- and broad-leaved trees (e.g., *Vachellia robusta*, *Senegalia nigrescens*, *Combretum* spp.) interspersed with patches of saline *Sporobolus* grassland, *Colophospermum mopane* woodland, sand forest, and riparian forest (Walker et al. 2023a, 2023b). Large mounds created by fungus-farming termites (Termitidae: Macrotermitinae) are abundant throughout the wooded habitats and support compositionally unique and spatially dispersed islands of woody vegetation (Tarnita et al. 2017, Daskin et al. 2023). On the escarpments, mixed savannas give way to miombo (*Brachystegia* and *Julbernardia* spp.) woodland and limestone-gorge forests, which cover 1,150 km$^2$ and 110 km$^2$, respectively (Lötter

et al. 2023; **Fig. 1a**). Riparian woodlands, floodplains, and wetlands (40 $km^2$) occur around several major perennial rivers, including the Pungue River, which runs along PNG's southern border, and the Urema River, which drains Lake Urema into the Pungue. Isolated inselberg woodlands (collectively around 10 $km^2$) occur on a handful of rocky outcrops (**Fig. 1a**). The vast majority of the data presented here were collected in the Rift Valley in the southwestern quadrant of PNG (**Fig. 2**), which encompasses the Urema floodplain, transitional savannas, and Rift Valley woodlands at the base and footslope of the western escarpment.

**d. Geology:** The geology and pedology of Gorongosa were described in detail by Tinley (1977), which is our primary source for this summary. The Rift Valley soils consist of sandy and clayey sediments that have been deposited by water and gravity to create gradients of soil texture and depositional age that map closely onto vegetation types. The floodplain soils are deep, fine, hydromorphic, anoxic vertisols. These clays are rich in organic matter, sodium, and other cations, have high extractable phosphorus content, and exhibit strong swell–shrink dynamics during seasonal waterlogging and drying. The saline soils underlying patches of mopane woodland and *Sporobolus* grassland are similar but coarser and lack the strong hydromorphic influence, leading to very high concentrations of exchangeable sodium and calcium. The alluvial and colluvial deposits underlying the Rift Valley woodlands are sandier and comparatively leached, with varying degrees of loam in the topsoil. The miombo woodlands of the western escarpment are underlain by shallow, sandy, nutrient-poor soils originating from Precambrian metamorphic rocks (gneiss); those on the eastern escarpment are underlain by limestone and sandstone. In all but the most intensively flooded areas, termites are important agents in regulating the texture, fertility, and spatial heterogeneity of surface and near-surface soils.

**e. Watersheds, hydrology:** PNG's central feature—geographically, hydrologically, and ecologically—is Lake Urema, which expands from a dry-season extent of $< 20$ $km^2$ to inundate up to 800 $km^2$ during the rains in January–February (**Fig. 1**). This flood cycle creates strong seasonal rhythms amongst the flora and fauna (Tinley 1977, Walker et al. 2023a). The shallow lake is fed by inflows from a 9,300-$km^2$ catchment comprising both sides of the Rift and Mount Gorongosa's orographic rainfall, which flows into the lake via the Vunduzi River. Mount Gorongosa also gives rise to several other significant rivers, including the Pungue, which merges with the Urema River

near the southeast corner of the park; this confluence forms the Dingedinge marsh, a seasonal 120-$km^2$ wetland. In addition to the perennial lake and rivers, PNG is dotted with seasonal pans—shallow depressions that fill with water during the wet season and gradually desiccate during the dry season (Tinley 1977). Some pans are big enough to retain water across multiple years, and even the more ephemeral ones are key waterholes during the dry season and exert a strong influence on animal distribution and space use (van Driessche et al. 2025).

**f. Site history:** Gorongosa was established as a game reserve in 1921 (1,000 $km^2$) and embiggened to 3,200 $km^2$ in 1935 before being designated as Mozambique's first national park in 1960. The area is an ancestral home of the Sena people, whose occupancy is thought to date back over 1,000 years, preceded by iron-age Bantu farming communities (Tinley 1977, Silva 2020). As of the mid-1900s, 33 species of large mammalian herbivores (≥ 5 kg) and 10 large carnivores, including lion (*Panthera leo*), leopard (*P. pardus*), hyena (*Crocuta crocuta*), and wild dog (*Lycaon pictus*), occurred in or near PNG. By the 1970s, several historically recorded species had been extirpated, including rhinos (*Ceratotherium simum*, *Diceros bicornis*), roan (*Hippotragus equinus*), tsessebe (*Damaliscus lunatus*), and cheetah (*Acinonyx jubatus*) (Tinley 1977). In 1972, the biomass of the nine largest herbivore species was estimated by fixed-wing surveys at roughly 9,000 kg $km^{-2}$ (Stalmans et al. 2019), including over 2,500 elephant (*Loxodonta africana*), 3,500 hippo (*Hippopotamus amphibius*), 13,000 buffalo (*Syncerus caffer*), 6,400 wildebeest (*Connochaetes taurinus*), 3,400 waterbuck (*Kobus ellipsiprymnus*), and 3,300 zebra (*Equus quagga crawshayi*).

PNG was contested during the Mozambican Civil War (1977–1992) between the government and insurgents based in the Gorongosa region. Heavy exploitation of PNG's wildlife throughout the war for food and sale, including ivory trafficking (Campbell-Staton et al. 2021), caused precipitous declines in large-mammal populations. Early post-war surveys (1994–1997), although limited in coverage, indicated that densities of the nine largest species had fallen by 93–99% (Stalmans et al. 2019; see also Daskin and Pringle 2018). The decline of large herbivores was accompanied by increases in woody cover throughout the Rift Valley between 1977 and 2012 (51–134%; Daskin et al. 2016), including proliferation of *Mimosa pigra*, an invasive shrub, in the floodplain (Guyton et al. 2020). Among large carnivores, wild dog, leopard, and hyena were all effectively extirpated; lions persisted but are thought to have declined by 90%. The remnant lion population consisted of

small prides that ate relatively small prey, chiefly warthog (Bouley et al. 2018, 2021). The collapse of apex predators and relaxation of predation risk led to ungulate behaviors and habitat affiliations that are rarely seen in other protected areas (Atkins et al. 2019). Similarly, the absence of large mammalian scavengers resulted in unusually slow rates of carcass decomposition (Phillips 2026).

Restoration efforts began in 1995 with an IUCN-led project that sought to rebuild infrastructure and employ park rangers (Morley & Convery 2014). A new phase of restoration began in 2008 with the establishment of the Gorongosa Restoration Project, a public-private co-management agreement between the Mozambican government and the Greg Carr Foundation (Pringle 2017, Pringle and Gonçalves 2022). Large-mammal populations have since rebounded via both natural recovery and selective translocations/reintroductions (Stalmans et al. 2019). By 2022, the biomass of the nine largest species had returned to pre-war levels (9,300 kg $km^{-2}$; Stalmans et al. 2022). The most recent helicopter-based total count in 2024 found that overall wildlife density and biomass were still increasing (Stalmans & Peel 2024). Experiments in the Lake Urema floodplain have shown that grazers now consume much of the grass and forb production (Becker et al. 2021), indicating the reestablishment of top-down control by large herbivores.

Nonetheless, community structure remains very different from the pre-war baseline (Stalmans et al. 2019, Stalmans & Peel 2024). Among the nine largest herbivores, the three biggest (elephant, hippo, buffalo) accounted for >86% of herbivore biomass before the war, compared with just 30% in 2024. In contrast, waterbuck accounted for <3% of pre-war biomass, compared with 66% in 2024 (Stalmans & Peel 2024). Sable (*Hippotragus niger*) and hartebeest (*Alcelaphus buselaphus lichtensteinii*) are relatively uncommon in many African savannas (Hatton et al. 2015); both initially increased in density during the post-war period, peaking between 2014–2018, but have since declined substantially (Stalmans & Peel 2024), possibly reflecting increased interspecific competition. Plains zebra, an historically abundant species in PNG that is also abundant in many other savannas (Hatton et al. 2015), has failed to increase from critically low abundance throughout the postwar period (mean population size of 33 from 2014–2024), despite the importation of 15 individuals from a hunting concession ~100 km northwest of PNG (Coutada 9).

Among species that were not enumerated in the pre-war period, several smaller ungulates (impala, *Aepyceros melampus*; warthog, *Phacochoerus africanus*; reedbuck, *Redunca arundinum*; oribi, *Ourebia ourebi*) proliferated rapidly during the post-war period. Data from biennial helicopter counts suggest that populations of these species are now either stable or declining, except for impala, which have continued to increase (Stalmans & Peel 2024).

Attempts to reestablish the historical large-carnivore community began around the same time that herbivore biomass was approaching pre-war levels. In 2018, a founding pack of 15 wild dogs (a globally endangered species) was reintroduced into Gorongosa from northwestern KwaZulu-Natal, South Africa (six free-ranging females from the Magudu area, nine males from iSimangaliso Wetland Park; Stalmans et al. 2019, Bouley et al. 2021). Over the next five years, 35 more wild dogs were imported from several source populations (15 from Khamab Kalahari Reserve, South Africa, in 2019; three from Tswalu Game Reserve and six from Hluhluwe-iMfolozi National Park and Lapalala Nature Reserve, South Africa, in 2021; and 11 from Maputo National Park, Mozambique, in 2023). As of 2024, the wild dog population appeared to have plateaued at around 250 individuals. Over the same period, small numbers of leopard, spotted hyena, and jackal (*Lupulella adusta*) were intermittently imported to PNG from elsewhere in Mozambique and neighboring countries. As of early 2026, the abundances of these species remained very low.

These events raise a number of general unanswered questions about community reassembly in the context of restoration and rewilding. Do communities develop along predictable successional pathways dictated by life-history traits (e.g., a shift in dominance from smaller to larger species), attracting community composition back towards a previous stable state (Deng et al. 2024)? Do the initial dominance and priority effects of a few early-recovering species (e.g., waterbuck) instead create an alternative stable state in which the recovery of formerly abundant species (e.g., large bulk grazers) is suppressed—perhaps even to the point of precluding stable coexistence by species such as zebra (Buschke et al. 2026)? Or, is the trajectory instead idiosyncratic and unpredictable owing to poorly documented initial conditions and the vagaries of chance events, such as extreme weather (e.g., Intense Tropical Cyclone Idai, which directly hit the park in 2019; Walker et al. 2023a)? Can we infer fluctuations in inter- and intraspecific competition—a notorious challenge in ungulate assemblages (Millar and Hickling 1990, Sinclair et al. 2003, Bagchi and Ritchie 2010,

Prins 2016)—from time-series data on population recovery, individual diets, and movements (Becker et al. 2021, Walker et al. 2023b, Anderson et al. 2024)? Similarly, what are the spatial and dietary signatures of intensifying predation and risk (Atkins et al. 2019), and how can the simultaneously shifting effects of top-down and bottom-up forcing be untangled? To what extent have severe bottlenecks influenced genetic diversity and caused Allee effects (e.g., in zebra)?

Monitoring Gorongosa's megafauna as it continues to reassemble can inform understanding of these and other key problems in population and community ecology in ways that are impossible at equilibrium, while also providing insights from the leading edge of large-mammal restoration at a time when conservation translocations are increasingly ubiquitous and efforts are underway to 'de-extinct' and rewild Pleistocene megafauna.

**g. Climate:** PNG has a tropical savanna climate (Köppen climate: Aw) with distinct wet and dry seasons. The wet season (austral summer, mean 27.1°C) is hotter than the dry (austral winter; mean 22.8°C; Tinley 1977). Most precipitation falls from November–April, leading to inundation of the floodplain (**Fig. 1d**). Total annual rainfall in the Rift Valley averages around 1,000 mm (IQR = 722–1,185 mm, from data collected in 1957–1969 and 2012–2024), and it is possible that the amount and/or variance of rainfall is increasing; 2018–2023 included four of the six wettest years on record while 2012–2017 had two of the driest (**Fig. 1c**). Rainfall increases with elevation from the valley to the escarpments (1,320 mm on the western Gorongosa plateau; 1,024 on the eastern Cheringoma Plateau) and Mt. Gorongosa (2,040 mm; Tinley 1977).

**h. Fire:** Fire is common in PNG, as in savannas worldwide (Staver et al. 2011), albeit with strong spatial and temporal heterogeneity (**Fig. 1b**). In a typical year from 2001–2023, 37% of the park burned (IQR = 28–47%). However, annual fire extent has roughly halved over that interval: 48% of PNG burned from 2001–2010 compared with only 26% from 2014–2023. It remains to be determined whether this trend reflects the recovery of grazing herbivores and consequent reduction of understory fuel. The park-wide mean fire-return interval (FRI) is 6.9 years, but FRI varies spatially from fewer than 2 years to over 15 years, with lower frequencies in floodplains (where plant biomass accumulation is limited) and in densely wooded areas, particularly on the eastern Rift escarpment.

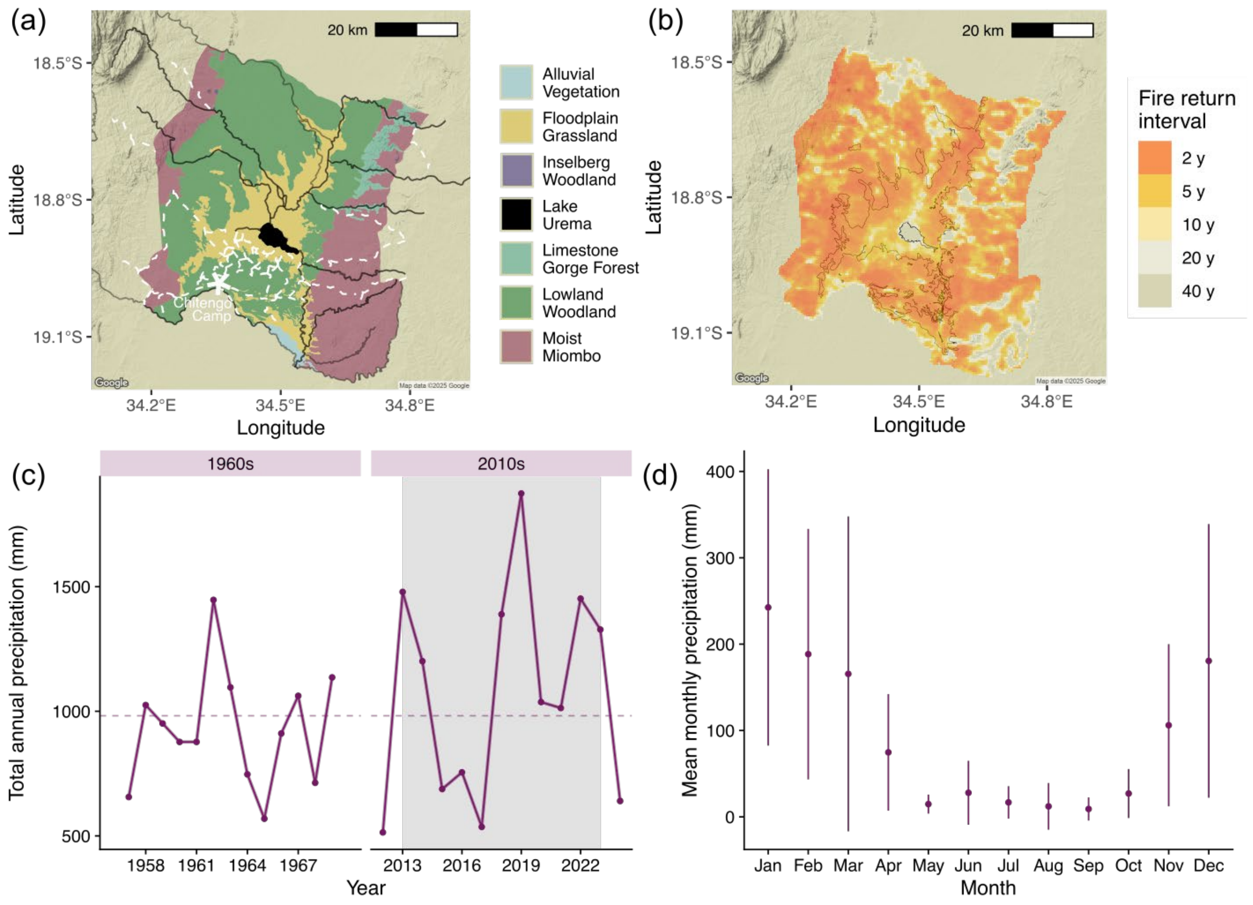


**Figure 1:** ***Geographic and bioclimatic features of Parque Nacional da Gorongosa (PNG). (a)*** *Seven major habitat types in Gorongosa: the largest being floodplain grassland (mustard yellow; 650 km$^2$), lowland woodland (green; 1,670 km$^2$), and miombo (magenta; 1,150 km$^2$), which are determined by the position of Lake Urema (black) and the eastern and western Rift escarpments. Vegetation boundaries are from Lötter et al. (2023); this map lumps three subtypes of miombo habitat and two subtypes of lowland savanna woodland habitat for cleaner visualization. Rivers are shown as solid black lines, roads as dashed white lines. A star shows the location of Chitengo, the administrative and research headquarters.* ***(b)*** *Fire-return interval (FRI) in PNG (excluding surrounding areas), calculated from the MODIS burned-area product (MCD64A1) for the period 1/1/2001 to 12/31/2023, shows that most of PNG burns at least once every few years. FRI for each 500-m$^2$ pixel was calculated as the number of years in the focal period (23 years) divided by the number of fires recorded in each pixel (pixels that never burned were assigned an FRI of 46 years). The floodplain habitat boundary is overlaid as a thin black line.* ***(c)*** *Field-measured annual precipitation in PNG (1957–1969 and 2012–2024) has ranged from 500–1,900 mm with a median of 980 mm (indicated by horizontal dashed line).* ***(d)*** *Mean monthly precipitation (2011–2024) shows strong seasonality, with a wet season from November–April and a dry season from May–October. Error bars (± 1 SD) show the large interannual variability in wet-season rainfall.*

**2. Sampling design**

**a. Rainfall monitoring:** Rainfall was monitored in Chitengo Camp (see **Fig. 1a**) by Kenneth Tinley (1957–1969), the local water authority (Ara-Centro; 1999–2005), researchers from Edinburgh University (2006–2009), and PNG's Department of Scientific Services (2011–2024). Some data are missing in the period 2005–2011, as discussed in *Class V.B.i*.

**b. Movement and space use:** Data on animal movements from GPS telemetry derive from several projects with different objectives and criteria for study-animal selection. Only individuals judged to be fully grown (based on body size, pelage, and/or horn growth) were selected for collaring. From 2014–2023, we captured 295 individuals of seven species and obtained location data on the movements of 277 unique individuals. This difference in sample size is a result of (a) 18 captures that yielded no GPS data owing to collar malfunctions, satellite communication failure, or mortality immediately after deployment (three bushbuck, two nyala); and (b) redeployment, in 2023, of GPS collars on seven kudu that had been collared in 2022 and were assigned the same unique animal identifier.

The majority of animals in these data (230 of 277, 83%) were collared as part of the Allometry of Spiral-Horned Antelopes: Movement Ecology and Diet (ASHAMED) project, which focuses on four sympatric species of spiral-horned antelope (*Tragelaphus* spp.) that collectively span a 10-fold range of adult body mass: bushbuck, nyala, kudu, and eland. In 2014 and 2015, respectively, we collared only female bushbuck ($n$ = 11 and 11), nyala ($n$ = 10 and 6), and kudu ($n$ = 12 and 10). In later years, we included both females (F) and males (M), with F:M ratios in parentheses:

2016: bushbuck (7:5)
2017: NA
2018: bushbuck (8:7), nyala (2:2), kudu (13:5)
2019: bushbuck (10:3), nyala (3:5), kudu (9:5)
2020: NA
2021: bushbuck (10:3), kudu (11:2)
2022: bushbuck (9:4), nyala (9:0), kudu (9:0), eland (4:0)
2023: bushbuck (11:4), kudu (4:0), eland (6:0)

For spiral-horned antelopes, we used GPS collars (2014–2016: Advanced Telemetry Systems G2110E iridium collar; 2018–2023: Vectronic Aerospace VERTEX Lite, with a 1D battery for female bushbuck and 3D batteries for all others) to record animals' locations in local time (UTC + 02:00). Mean GPS measurement error for these collars in PNG is approximately 13 m (Atkins et al. 2019). Our default fix rate was 1 h, but we sometimes increased it up to a maximum of every 3 min depending on battery life and the needs of specific studies (Daskin et al. 2023, Walker et al. 2023a). Collar-belt circumferences differed among species and sexes (bushbuck: F = 25.5 cm, M = 38 cm; nyala: F = 35.5 cm, M = 53.25 cm; kudu: F = 43 cm, M = 79 cm; eland, F = 90 cm).

The remainder of the movement data are from 47 individuals of three species: waterbuck, elephant, and zebra. In 2015 and 2016, we captured 30 female waterbuck and deployed custom-built collars outfitted with GPS units, tri-axial accelerometers, and video cameras ('CritterCams'; National Geographic Labs, DC, USA). The collar design is described in detail in Park et al. (2019), and data are reported in Becker et al. (2021). Of these collars, 22 produced usable location data over a median duration of 13 days (range 2.9–22.8 d). These collars were designed to adaptively alter the GPS-fix rate as follows: every 600 s when the animal was at rest, every 400 s during activity, and every 20 s when other collared waterbuck were within 40 m (Becker et al. 2021).

Elephants were collared in 2015 (0:6), 2016 (0:6), and 2018 (6:0) to study (a) human-wildlife conflict involving cross-boundary movements between PNG and surrounding farmlands (Branco et al. 2019, 2020, Gonçalves 2023), and (b) potential differences in the ecology of tusked and congenitally tuskless female elephants (Campbell-Staton et al. 2021). The collars deployed in 2015 and 2016 (Africa Wildlife Tracking, AWT IM-SAT; South Africa) lasted approximately two years and recorded locations every 30 minutes. Those deployed in 2018 (Savanna Tracking Savanna Elephant Iridium Collars, South Africa; 4D battery, 400 cm belt circumference trimmed to fit each individual) lasted approximately three years and recorded locations every 15 minutes. An additional and more extensive set of elephant GPS-collar data collected by D.D.G. is not included here.

Seven zebras were collared in 2022 (2:2) and 2023 (1:2) for insight into the behavior of this extremely low-density population and the possible reasons for its failure to increase from low abundance up to 2024. These collars (Vectronic Aerospace VERTEX Lite; 3D battery, 75 cm belt circumference) were programmed to record locations every 30 minutes and were collected after one year. Although these sample sizes are small, they represent nearly 10% of the total estimated zebra population.

**c. Diet analysis:** We began collecting herbivore fecal samples for DNA metabarcoding in 2013. Focal species, frequency, spatiotemporal scale of collection, and intensity of sampling varied across years depending on the needs of specific projects. Most samples were collected by driving PNG's road network (**Fig. 2A**) and watching for defecating animals. A smaller subset of samples was collected from animals that were chemically immobilized for GPS collaring, where we extracted samples directly from the rectum (see *II.B.3.a.ii* for capture methods and protocols).

Upon collection, each sample was assigned a categorical value describing the condition of the sample, which we used to calibrate our confidence in the recorded species identity and the potential for environmental contamination. These categories were: 5 = sample was taken directly from rectum of an animal immobilized for GPS collaring (least potential for misidentification and contamination); 4 = observers witnessed defecation and collected samples immediately thereafter; 3 = fresh dung was found and the purported species of origin was seen in the immediate vicinity; 2 = fresh dung was found but the purported species of origin was not seen in the immediate vicinity; 1 = sample condition was not recorded (applying to less than 4% of all samples, 142 of 3,785). For common species (e.g., waterbuck) and those with dung that is difficult to distinguish (e.g., the similarly sized antelopes impala, bushbuck, reedbuck, and nyala), we were more selective and tried to limit our collections to samples in categories 4 and 5. For rarely encountered species (e.g., zebra, eland, hippo, duiker) and moderately common species with easily identifiable dung (e.g., elephant), we would generally accept samples of any category.

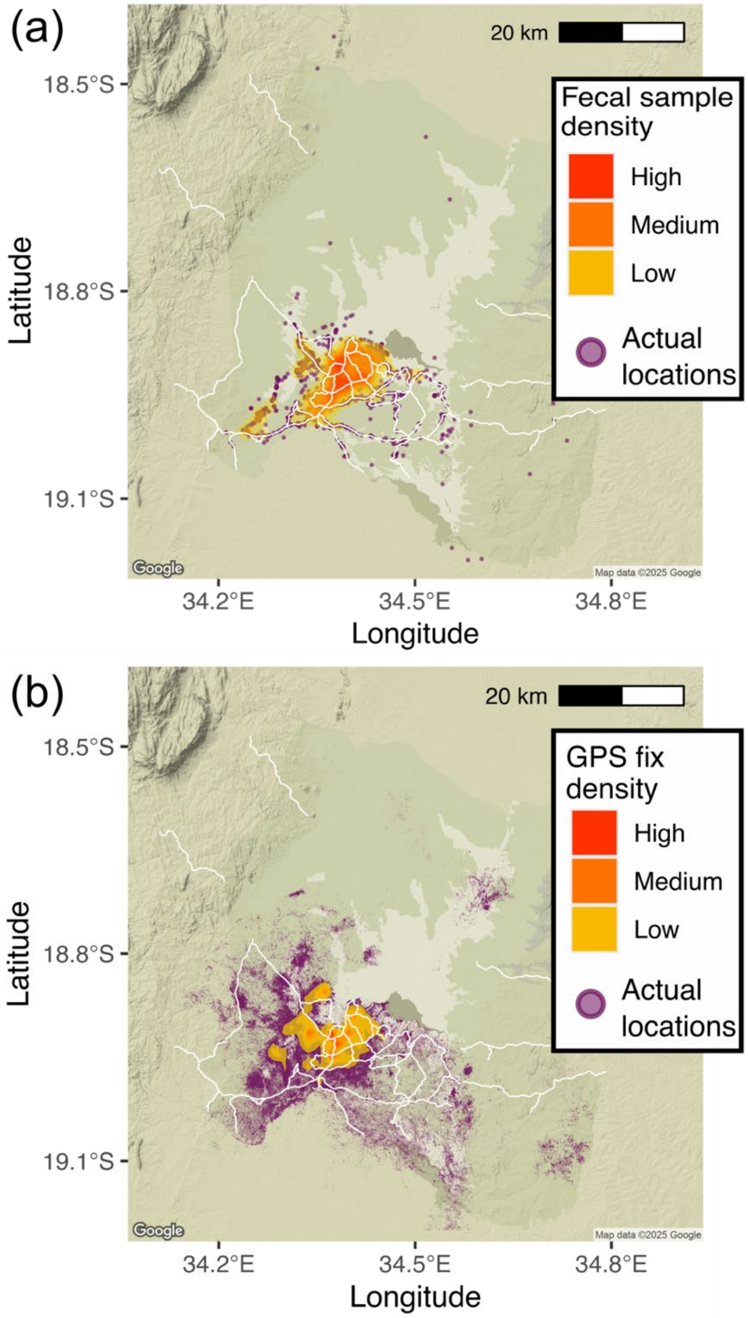


***Figure 2: Spatial distributions of fecal-sample collections and GPS-collar locations. (a)** Density distribution (yellow-to-red gradient) and actual locations (purple points) of fecal samples collected for DNA metabarcoding superimposed on a map of PNG (see **Fig. 1a**). Fecal samples were primarily collected adjacent to dirt roads (white lines) in the southwestern quadrant of the park. **(b)** Density distribution and actual locations of 1.7 million randomly selected GPS fixes (roughly 50% of the total) show the intensity and extent of space use. The most intensively used areas overlap heavily with fecal-sample locations (as seen by comparing the density distributions).*

Overall, the greatest intensity of fecal sampling was in the early dry season (June–August of 2013–2019 and 2021–2022; $n = 2{,}426$). However, we also present data from sizeable numbers of samples collected during the late wet (March–May of 2017–2020 and 2022; $n = 712$) and late dry seasons (September–November of 2016–2019 and 2021; $n = 647$). From 2013–2020, sampling occurred during season-specific expeditions. In 2021–2022, sampling was continuous from July–November 2021 and April–July 2022.

As an idealized baseline, we aimed to collect at least 15 fecal samples per species during a given sampling period. Actual sample sizes were often much larger than this for abundant species and/or when projects required intensive sampling of certain species (e.g., waterbuck and buffalo in 2015: Guyton et al. 2020, Becker et al. 2021; bushbuck in 2018–2019: Atkins et al. 2019, Walker et al. 2023b, van Driessche et al. 2025). Conversely, sample sizes were limited for a suite of rarely encountered ungulates (zebra, eland, bushpig, red duiker, hippo). We opportunistically sampled several non-ungulate species, including substantial numbers of chacma baboon (*Papio ursinus*) and Cape porcupine (*Hystrix africaeaustralis*) along with small numbers of a half-dozen carnivore species. For all species, however, we analyzed only the plant component of the diet.

Our earliest work involved small numbers of species and samples. Over time, we expanded the scope to include the entire large-herbivore community and correspondingly larger sample sizes:

2013: waterbuck and warthog only (total $n = 70$)
2014: bushbuck, nyala, and kudu only (total $n = 99$)
2015: 10 species, including 6 with $n \geq 10$ samples (total $n = 321$)
2016: 20 species, including 15 with $n \geq 10$ samples (total $n = 325$)
2017: 16 species, including 6 with $n \geq 10$ samples (total $n = 272$)
2018: 18 species, including 16 with $n \geq 10$ samples (total $n = 882$)
2019: 19 species, including 16 with $n \geq 10$ samples (total $n = 776$)
2020: 12 species, including 6 with $n \geq 10$ samples (total $n = 111$, interrupted by a pandemic)
2021: 18 species, including 14 with $n \geq 10$ samples (total $n = 527$)
2022: 17 species, including 12 with $n \geq 10$ samples (total $n = 402$)

## 3. Research methods

### a–b. Field/laboratory methods and instrumentation

**i. Rainfall data:** Rainfall was monitored with a manual rain gauge in Chitengo from 1957–1969 and 1999–2024. In 2015, an automated gauge was also installed. A TAHMO Weather Station (ATMOS 41, METER Group, WA, USA) was used from 2015–2023 and then replaced with a Davis Weather Station (Vital Weather, South Africa). Since 2015, rainfall records have been based primarily on data from the automated station. Readings from the manual gauge are collected concurrently for validation and for backup when the main gauge fails.

**ii. GPS collaring and animal measurements:** Animals were immobilized using species-specific mixes and doses of thiafentanil (etorphine for zebra), medetomidine, azaperone, and ketamine. We used naltrexone to reverse the opioids and atipamezole to reverse medetomidine. When darting from a vehicle, we used transmitter darts to track and recover immobilized individuals. Animal-care protocols and permits are listed in *Class II.B.3.e: Legal and organizational requirements*.

***Spiral-horned antelopes.*** Processing of *Tragelaphus* spp. (bushbuck, nyala, kudu, and eland) involved fitting GPS collars, collecting fecal, hair, blood, and horn samples, and recording body measurements. We collected feces from the rectum while wearing nitrile gloves, plucked hair samples from the tail or rump, and drew blood from the jugular or cephalic veins. Horn samples ($\geq$ 0.5 mg) were collected for isotopic analysis of resource use through time, using a Dremel Micro 8050-35 rotary tool with a 1.6-mm bit, at predetermined intervals along the leading spiral edge of the right horn (bushbuck: 4, 15, 25, 35, 38, 39 cm; nyala: 8, 23, 38, 52, 62 cm; kudu: 4, 24, 41, 64, 76, 91, 109, 127 cm). These intervals were chosen based on literature records of tragelaphine horn growth rates (Simpson 1966, 1971, 1972, Anderson 1986, Furstenburg 2010, 2016). Body length, hind-foot length, and chest girth were measured with a tape. For bushbuck and female nyala, we weighed individuals on a spring scale (nearest 0.1 kg). Body mass estimates for male nyala and all kudu were extrapolated from a linear regression between the measured chest girths and body masses of female bushbuck and female nyala captured in 2014, which accounts for the weight of the bag and GPS collar ($n$ = 22, $R^2$ = 0.91):

$$\boldsymbol{M} = 1.604\boldsymbol{c} - 84.004$$

where $M$ is body mass in kg and $c$ is chest girth in cm (Daskin et al. 2023). We present measured and estimated body-mass values separately and advise cautious interpretation of the latter. We used a portable ultrasound machine (measurement precision = 1 mm; E.I. Medical Imaging Ibex Pro; CO, USA) to measure maximum rump-fat depth, thickness of the biceps femoris and longissimus dorsi muscles, and pregnancy status in females. We also recorded standardized body-condition scores based on palpation (by R.A.L.) of the sacrosciatic ligament, lumbar vertebrae, sacrum, base of tail, and caudal vertebrae. Udder palpation was used to assess lactation status in females. Subsets of these data, along with additional methodological details, are included in published studies (Atkins et al. 2019, Daskin et al. 2023, Walker et al. 2023a, 2023b, van Driessche et al. 2025).

***Other species.*** Field methods varied for other GPS-collared species (waterbuck, elephant, zebra). Waterbuck were immobilized with a combination of thiafentanil, medetomidine, and azaperone. We recorded body measurements as for spiral-horned antelopes and fitted each waterbuck with a custom-built collar (Park et al. 2019; Becker et al. 2021). Elephants were immobilized using combinations of thiafentanil (9–15 mg) and azaperone (40–60 mg), with dosage based on estimated age and size. Ground teams located each animal following immobilization and monitored vital rates during GPS-collar fitting. We collected fecal and blood samples but did not record morphometric measurements (Branco et al. 2019, 2020, Campbell-Staton et al. 2021). Zebras were immobilized with etorphine, medetomidine, and azaperone. We collected fecal, hair, and blood samples and measured body length, chest girth, and hind-foot length while GPS collars were being fitted.

**iii. Fecal DNA metabarcoding:** Field and laboratory procedures for DNA metabarcoding have been largely consistent, especially since 2016, as described in publications that have used the data (Atkins et al. 2019, Branco et al. 2019, Pansu et al. 2019, 2022, Guyton et al. 2020, Becker et al. 2021, Potter et al. 2022, Daskin et al. 2023, Walker et al. 2023a, 2023b, van Driessche et al. 2025).

***Field collection.*** Upon discovery of fresh feces, the collector, wearing an unused nitrile glove, pinched off portions of upwards-facing dung with no visible contamination from soil, vegetation, invertebrates, or disturbance and placed them in an unused plastic zip bag. This process was

repeated until at least 15 mL of dung had been collected, or until no more uncontaminated dung remained. Dung taken from immobilized animals was bagged immediately; in cases when little to no feces were present in the rectum, the collector transferred any fecal fragments and mucus from the exterior of the glove to the interior of the bag. We then sealed the bag, labeled it with identifying information (species and sample number), and recorded metadata (GPS location, habitat type, date, time, sex, notes) on a datasheet. Bagged samples were stored in an insulated container with ice packs for at most a few hours until we returned to the field laboratory at Chitengo.

***Pre-processing in the field.*** Fecal samples were pre-processed at the field laboratory to preserve DNA for transport. Bagged samples were manually massaged to homogenize the sample as much as possible. We transferred a pea-sized subsample (roughly 200 $mm^3$) of homogenized sample from the collection bag to a 2-mL tube that had been pre-filled with (a) glass beads and 540 µL of SLX-Mlus buffer (2013; Omega Biotek, CO, USA) or (b) silica beads and 750 µL of DNA lysis and preservation buffer (2014–2022; Xpedition Lysis/Stabilization Solution; Zymo Research, CA, USA). Tubes were labeled, capped, and mixed for 30 s using a flat-bed vortex to break apart the feces and expose DNA to buffer solution. Before export to the United States for DNA extraction, amplification, and metabarcoding (see *II.B.3.e* for permitting information), we subjected all samples to antiviral treatments mandated by the US Department of Agriculture. In 2013–2015, this entailed application of proteinase K, heating to 95°C for 15 min, and treatment with RNase A. Upon issuance of revised regulations, we switched to a heat treatment of 72°C for 30 min. Samples were frozen at -20°C prior to transport. On arrival at Princeton University, samples were stored at -80°C. Leftover sample and extracts remain stored indefinitely at -80°C in R.M.P.'s laboratory.

***Sample preparation and sequencing.*** We usually processed samples within 2 mo. of collection. Thus, samples from different collection periods were processed independently. DNA was extracted using EZNA Stool DNA kits (2013; Omega Biotek, GA, USA) or Zymo Quick-DNA Soil/Fecal Microbe MiniPrep kits (2014–2022) following manufacturer protocols. Extractions were generally conducted in batches of 29 samples and one negative extraction control containing only DNA lysis buffer. These samples were sorted onto 96-well plates in chronological order, except when chronological ordering would have filled an entire plate column or row samples from a single species; in those cases, samples were shuffled across plates.

We amplified DNA from the P6 loop of the chloroplast *trn*L (UAA) intron (Kartzinel et al. 2015, Pansu et al. 2019, 2022) with the *g* (forward: 5′-GGGCAATCCTGAGCCAA-3′) and *h* (reverse: 5′-CCATTGAGTCTCTGCACCTATC-3′) primers (Taberlet et al. 2007). Primers were dual indexed using unique tags (8 nt, with a minimum of four differences between tags) at the 5' end of each primer, which enabled multiplexing of PCR products in a single sequencing library. From 2018–2020, we standardized DNA concentrations per sample prior to PCR amplification using the Quant-iT PicoGreen assay (ThermoFisher Scientific, MA, USA) and nucleic-acid free water (Qiagen, MD, USA). From 2015 onwards, we performed PCRs in triplicate and always included negative extraction controls and negative PCR controls (nucleic acid-free water). We also included positive PCR controls for samples collected in 2013–2017 and 2021–2022 (Pansu et al. 2022). PCR mixtures contained 1X GeneAmp PCR buffer II (Applied Biosystems, MA, USA); $MgCl_2$ at 2.5 mM (Applied Biosystems, MA); dNTPs at 0.2 mM (New England BioLabs, MA, USA); 0.17 mg $ml^{-1}$ of BSA (New England Biolabs); each primer at 0.2 μM; 0.4% dimethyl sulfoxide (Sigma-Aldrich, MO, USA); 0.5 U AmpliTaq Gold DNA Polymerase (Applied Biosystems); and 2 μL of DNA extract in a final reaction volume of 20 μL (in 2013 only, the same reagents were used in a reaction volume of 12.5 μL: Guyton et al. 2020). Reactions consisted of initial denaturing (95°C; 10 min), 35 cycles of denaturing (95°C; 30 s), annealing (55°C; 30 s), and extension (72°C; 30 s), and final elongation (72°C; 2 min). We used gel electrophoresis to verify amplification of the P6 marker in haphazardly selected subsets of samples and positive controls (and the lack thereof in negative controls).

PCR products were then pooled for purification. From 2013–2015, PCR products were first standardized (SequelPrep Normalization Kit, Applied Biosystems) and then purified with Zymo Clean and Concentrator kits; from 2016–2022, they were purified using Qiagen MinElute PCR Purification kits. Purified products were pooled into equimolar sequencing libraries. Illumina tags were appended to sequencing libraries with a low-cycle PCR approach (2013–2015 and 2018–2022) or a PCR-free approach (2016–2017) at Princeton's core facility. The size distribution of the purified products was assessed by facility staff using a bioanalyzer. From 2015–2022, we obtained 2×150 base pair (bp) paired-end sequence data generated on Illumina platforms, including HiSeq 2500 (2013–2017, early dry season 2018), MiSeq 600-nt (late wet season 2018-2019, late

dry season 2018–2019, all seasons 2021–2022), and NovaSeq SP 300-nt (2020); in 2013 and 2014, and for the subset of *Tragelaphus* spp. samples from 2015, we obtained 170-bp single-end reads (Guyton et al. 2020, Daskin et al. 2023).

When defecation was not directly observed and dung morphology was inconclusive, we verified herbivore species identity by amplifying and sequencing mammalian DNA in the fecal sample. In all, we analyzed mammal DNA for 1,167 of the 3,295 samples collected from 2016–2022. We amplified DNA from the mitochondrial 16S gene using the MamP007 primer pair: MamP007F, 5'-CGAGAAGACCCTATGGAGCT-3'; MamP007R, 5'-CCGAGGTCRCCCCAACC-3' (Giguet-Covex et al. 2014). Primers were tagged and multiplexed as described for plant metabarcoding. PCR mixtures contained 1X GeneAmp PCR Buffer II; $MgCl_2$ at 2mM; dNTPs at 0.2mM; 0.2 µg BSA; each primer at 0.2µM; 0.5U of AmpliTaq Gold DNA Polymerase; and 2 µL of DNA extract in a final reaction volume of 20 µL. Reactions consisted of initial denaturing (95°C; 10 min), 45 cycles of denaturing (95°C; 30 s), annealing (52°C; 30 s), and extension (72°C; 30 s), and final elongation (72°C; 7 min). Pooled PCR products were purified using MinElute PCR Purification kits and combined into equimolar sequencing libraries. Libraries were prepared using a low-cycle PCR approach, amplicon size distribution was confirmed using a bioanalyzer, and libraries were sequenced (2×150 bp paired-end) on a NovaSeq SP 300-nt (2016–2020), MiSeq 600-nt (early 2021), or MiSeq Micro 300-nt (late 2021, 2022).

After sequencing, we applied a comprehensive bioinformatic filtering process to all sequence data. Our protocol is detailed in multiple previous publications (Atkins et al. 2019, Pansu et al. 2019, Guyton et al. 2020, Becker et al. 2021, Pansu et al. 2022, Potter et al. 2022, Walker et al. 2023a, Walker et al. 2023b, van Driessche et al. 2025) and in *Class V.D.iii*. Briefly, we first used the OBITools filtering pipeline (Boyer et al. 2016) to align paired-end reads, associate sequences with their sample of origin, remove putative errors and sequences with low read counts, assign taxonomic identifiers, and evaluate sequence variants. Next, we used R to refine taxonomic assignments, identify and remove putative contaminant and chimeric sequences, remove PCR replicates with low sequencing depth, and assess consistency among PCR replicates. To confirm herbivore species identity, we used the same approach to data filtering before applying the decision framework described by Pansu et al. (2022) to identify the species of origin. Last, we merged all

data sets after harmonizing sample-naming and metadata conventions, correcting species identity where necessary based on the mammal DNA analysis, and refining the taxonomic assignment of the most common sequences using botanical inventories for Gorongosa. For a full description of bioinformatic filtering methods, refer to *Class V.D.iii*.

**c. Taxonomy and systematics:** Plant DNA found in large-mammalian herbivore fecal samples was matched to reference sequences of the *trn*L (UAA) intron extracted from plant specimens collected in PNG and deposited locally in the herbarium collection at Chitengo. This set of reference sequences is deposited with Pansu et al. (2019) and is also included here (data set *20*). Botanical nomenclature in data sets *11–12* follows the conventions of Kew Botanical Garden's Plants of the World Online database (https://powo.science.kew.org/). We follow Hassanin et al. (2018) in considering bushbuck in PNG to be *Tragelaphus sylvaticus* and eland to belong in *Tragelaphus* (*T. oryx*) rather than *Taurotragus*; note, however, that the codes we use for these species in the deposited data sets reflect prior taxonomic understanding (i.e., bushbuck are coded as 'TRSC' and eland as 'TOAR', rather than 'TRSY' and 'TROR', respectively).

**d. Permit history:** All fieldwork was conducted under permits issued by PNG's Department of Scientific Services (e.g., PNG/DSCi/C92/2018; PNG/DSCi/C140/2019; PNG/DSCi/C190/2021; PNG/DSCi/C223/2022; PNG/DSCi/C291/2023; PNG/DSCi/C298/2023; PNG/DSCi/C329/2024).

**e. Legal and organizational requirements:** Animals were immobilized according to protocols approved by the Institutional Animal Care and Use Committees of Princeton University (1958-13, 2075F-16) and the University of Idaho (2019-32, 2015-39) and followed guidelines endorsed by the American Society of Mammalogists (Sikes and Animal Care and Use Committee of the American Society of Mammalogists 2016). These protocols were also approved by PNG's Department of Conservation. Fecal samples were imported to the United States and transported to Princeton University in accordance with permits issued to R.M.P. by the US Department of Agriculture's (USDA) Animal and Plant Health Inspection Service (APHIS; permit numbers 122489, 123156, 130123, 639-23-151-92157) and were stored and analyzed in R.M.P's certified Biosafety Level 2 laboratory. Sample export from Mozambique was authorized by the Provincial Directorate of Agriculture and Fisheries within Mozambique's Department of Livestock.

**4. Project personnel:** The leadership, staff, and research community of Gorongosa National Park, past and present, contributed to this work in countless vital ways. We thank Park Wardens Mateus Matemba and Pedro Muagura for their support and oversight. PNG's Department of Conservation provided veterinary services for animal captures, operational support, and access to information and resources, with special thanks to Ângelo Levi, Rui Branco, Mércia Angela, and Antonio Paulo (Tonecas). PNG's Department of Scientific Services was invaluable in enabling all facets of this work; we thank Francesca Andicene, Flavio Artur, Ana Gledis da Conceição, Jason Denlinger, Miguel Lajas, and Margarida Victor. External pilots and wildlife professionals provided crucial assistance with animal captures: Harry Hensberg, Benjamin Osmers, Mike Pingo, and Louis van Wyk. In addition to the authors, other investigators in PNG contributed to the collection of these data, both directly and indirectly: Brian Arnold, Paola Bouley, Paola Branco, Susana Carvalho, Tyler Coverdale, Shane Campbell-Staton, Joshua Daskin, Kaitlyn Gaynor, Jennifer Guyton, Tyler Kartzinel, Tara Massad, Kevin Monteith, Meredith Palmer, Jeremy Van Driessche, and Bart Wursten, among others. Undergraduate students from Princeton University and the University of Idaho also participated in fieldwork, including Courtney Buoncore, Megan Demmel, Madeleine Dietrich, Katie Grabowski, Luca Kuziel, Alana Reynolds, Alexander Getraer, Elyce Gosselin, Catherine Keim, Vienna Lunking, Stephanie Neatby, Matthew Rafferty, Zoe Rennie, Savannah Rogers, Maria Stahl, and Bobo Stankovic.

**Class III. Data set status and accessibility**

**A. Status**

**1. Latest update:** November 2025

**2. Latest archive date:** November 2025

**3. Metadata status:** The metadata are current and stored with the data.

**4. Data verification:** All archived data were verified by M.C.H. and R.H.W.

**B. Accessibility**

**1. Storage location and medium:** Filtered data, metadata, and data-cleaning scripts are provided in Data S1 and on Dryad (https://doi.org/10.5061/dryad.m905qfv85). Movement data are also

deposited on Movebank (Study ID#: 7359354918). Raw DNA sequencing data (data sets *18* and *19* in Metadata S1) are omitted from Data S1 due to their large size and are instead only provided in the Dryad repository. Fecal DNA metabarcoding data are also stored on the high-performance computing clusters at Princeton University and the University of California, Merced, and raw GPS-collar data are stored on OneDrive at the University of Idaho.

**2. Contact persons:** Robert M. Pringle, Department of Ecology and Evolutionary Biology, Princeton University (email: rpringle@princeton.edu); Ryan A. Long, Department of Fish and Wildlife Sciences, University of Idaho (email: ralong@uidaho.edu).

**3. Copyright restrictions:** None

**4. Proprietary restrictions:** Our research has been supported by public funds, and the data are freely available for public use. We do not stipulate any restrictions on the use of these data aside from citation of this paper and, if relevant, any previously published works based on these data. As matters of prudence and courtesy, however, we request advance notification of plans to make use of the data—in part to identify potential synergies among research efforts, in part so that we can supply additional guidance or caveats that might be relevant to a given project, and in part because these data are part of continuing long-term research and are thus continually being augmented. For example, fecal samples collected from 2023–2025 were being analyzed at the time of writing and could not be included here, and we plan to continue collecting for the foreseeable future; the same is true for the GPS-telemetry data. We also request notification upon the publication of any work that uses these data, so that we can report outputs to funders and other interested parties.

**a. Release date:** Data for this publication were made available on Dryad (DOI: 10.5061/dryad.m905qfv85) on March 4, 2026.

**b. Citation:** Users should cite this data paper when using the data.

**c. Disclaimers:** None.

**5. Costs:** None.

**Class IV. Data structural descriptors**

Reference key for the four-letter codes used to denote mammal species identity in the data sets.

| **Code** | **Latin name** | **Common name** |
|---|---|---|
| KOEL | *Kobus ellipsiprymnus* | Waterbuck |
| PHAF | *Phacochoerus africanus* | Common warthog |
| TRSC | *Tragelaphus sylvaticus* | Cape bushbuck (imbabala) |
| TRAN | *Tragelaphus angasii* | Nyala |
| TRST | *Tragelaphus strepsiceros* | Greater kudu |
| TAOR | *Tragelaphus oryx* | Common eland |
| AEME | *Aepyceros melampus* | Impala |
| OUOU | *Ourebia ourebi* | Oribi |
| POLA | *Potamochoerus larvatus* | Bushpig |
| REAR | *Redunca arundinum* | Southern reedbuck |
| SYCA | *Syncerus caffer* | Cape buffalo |
| ALBU | *Alcelaphus buselaphus lichtensteinii* | Hartebeest (Lichtenstein's subspecies) |
| CENA | *Cephalophus natalensis* | Red duiker |
| CHPY | *Chlorocebus pygerythrus* | Vervet monkey |
| CICI | *Civettictis civetta* | African civet |
| COTA | *Connochaetes taurinus* | Blue wildebeest |
| EQQU | *Equus quagga crawshayi* | Plains zebra (Crawshay's subspecies) |
| HINI | *Hippotragus niger* | Sable antelope |
| LESE | *Leptailurus serval* | Serval |
| LOAF | *Loxodonta africana* | Savanna elephant |
| PAUR | *Papio ursinus* | Chacma baboon |
| ATPA | *Atilax paludinosus* | Marsh mongoose |
| HEIC | *Herpestes ichneumon* | Egyptian mongoose |
| HIAM | *Hippopotamus amphibius* | Hippopotamus |
| PALE | *Panthera leo* | Lion |
| SMTE | *Smutsia temminckii* | Ground pangolin |
| HYAF | *Hystrix africaeaustralis* | Cape porcupine |
| UNKN | NA | Unknown species |

## 1. ANNUAL RAINFALL DATA FOR GORONGOSA

### A. Data set file

**Identity:** annual_rainfall_1957-2024.csv

**Size:** 0.5 KB

**Format:** CSV

**Contents:** Annual rainfall measured at Chitengo, PNG's administrative headquarters, between 1957 and 2024. There are 39 lines of data, each containing the year and total rainfall in millimeters.

### B. Variable information

| Column | Attribute | Definition |
|---|---|---|
| 1 | Year | Calendar year for which rainfall is reported. |
| 2 | Rainfall (mm) | Total rainfall accumulated during the year in millimeters. |

**C. Data anomalies:** Rainfall data are lacking for 1970–1998 and 2010–2011. In 2005 and 2009, measurements were not recorded continuously for the entire year. In 2005, data are missing for November (typically a semi-dry month; Fig. 1d); in 2009, data are missing for May–July (typically among the driest months; Fig. 1d) and for December (typically the onset of the wet season; Fig. 1d). In addition, we consider the recorded total for 2008 (204 mm) to be unrealistically low. Data for these three years should be interpreted accordingly. Data from the period encompassing the movement and diet data (2013–2024) are complete and reliable, and from 2015 onwards, were measured using an automatic weather station and cross-validated with a manual rain gauge.

## 2. MONTHLY RAINFALL DATA FOR GORONGOSA

### A. Data set file

**Identity:** monthly_rainfall_2011-2024.csv

**Size:** 3 KB

**Format:** CSV

**Contents:** Monthly rainfall from October 2011–December 2024 in Chitengo. There are 159 lines of data, each containing the year, month, and total rainfall in millimeters.

### B. Variable information

| Column | Attribute | Definition |
|---|---|---|
| 1 | Year | Calendar year for which rainfall is reported. |
| 2 | Month | Calendar month for which rainfall is reported. |
| 3 | Rainfall (mm) | Total rainfall accumulated during the month in millimeters. |

**C. Data anomalies:** None.

## 3. COLLAR, CAPTURE, AND FATE DATA

### A. Data set file

**Identity:** COLLAR-CAPTURE-AND-FATE-RECORDS_2014-2023.csv

**Size:** 71 KB

**Format:** CSV

**Contents:** Metadata for movement data sets: animal identifiers, date and location of capture, dates of first and last GPS fixes, body measurements, tissues sampled, and fate (mortality or survival) of all captured animals. There are 302 lines of data, each corresponding to one captured animal.

### B. Variable information

| Column | Attribute | Definition |
|---|---|---|
| 1 | AnimalID | Unique individual ID (13–17 characters), including species code, year of collar deployment, sex (f, female; m, male), collar serial ID, and intra-annual collar deployment number (0 = not recorded; 1 = first individual on which that GPS collar was deployed in that year; 2 = second individual on which that collar was deployed that year). |
| 2 | Species | Species common name |
| 3 | Sex | Female or male |
| 4 | CollarSerial | Unique identifier (serial number) for the deployed collar. |
| 5 | Latitude | Latitude of the capture location (WGS1984). |
| 6 | Longitude | Longitude of the capture location (WGS1984). |
| 7 | DeployDate | Calendar date of capture in YYYY-MM-DD format. |
| 8 | DeployYear | Year of capture in YYYY format. |
| 9 | DeployMonth | Month of capture written long-form. |
| 10 | DeployDayOfYear | Days from January 1st of the capture year to the capture date. |

| | | |
|---|---|---|
| 11 | FirstFixDate | Date of first GPS collar location in YYYY-MM-DD format. |
| 12 | LastFixDate | Date of last GPS collar location in YYYY-MM-DD format. |
| 13 | Fetus | Confirmed presence of a fetus (1) or no fetus detected (0). |
| 14 | Lactate | Confirmed lactating female (1) or no lactation detected (0). |
| 15 | MaxFat_mm | Maximum rump-fat depth measured using ultrasound (mm). Values reported as "<1" indicate fat was present but not measurable given the ultrasound's measurement precision (1 mm). |
| 16 | Bfemoris_mm | Thickness of biceps femoris muscle measured using ultrasound (mm). |
| 17 | Ldorsi_mm | Thickness of longissimus dorsi muscle measured using ultrasound (mm). |
| 18 | SSligament_score | Palpation score at the sacrosciatic ligament (0–6). |
| 19 | Lumbar_score | Palpation score at the lumbar vertebrae (0–6). |
| 20 | Sacrum_score | Palpation score at the sacrum (0–6). |
| 21 | BaseTail_score | Palpation score at the base of the tail (0–6). |
| 22 | Caudal_score | Palpation score at the caudal vertebrae (0–6). |
| 23 | ChestGirth_cm | Circumference of chest at the withers (cm). For the largest ungulates (eland, zebra), we typically measured half the chest girth and multiplied by 2. |
| 24 | BodyLength_cm | Dorsal length from nose to base of the tail (cm). |
| 25 | HindFoot_cm | Length of hind foot from hoof tip to hock (cm). |
| 26 | MeasuredWeight_kg | Body mass (kg) measured with a portable scale in the field. |
| 27 | EstimatedWeight_kg | Body mass (kg) extrapolated from a regression equation. As male nyala and all kudu are too heavy to lift, we have previously predicted their body masses using a linear regression based on chest girth, fitted using data from bushbuck and female nyala that were weighed in the field in 2014: $Mass = 1.604 \times Chest\ girth - 84.004$. We present |

| | | |
|---|---|---|
| | | estimates for all bushbuck, nyala, and kudu, including those that were weighed, but not for other species, as the regression is unlikely generalizable across other body plans. These estimates are all highly plausible but should be interpreted cautiously as extrapolations beyond the data. |
| 28 | FecalSample | Whether a fecal sample from the individual produced usable dietary data (1) or not (0). |
| 29 | FecalDietID | Fecal sample identifier in the dietary data sets ("Sample_ID" in data sets *11–13*). |
| 30 | HairSample | Whether a hair sample was collected (1) or not (0). |
| 31 | BloodSample | Whether a blood sample was collected (1) or not (0). |
| 32 | HornSample | Whether a horn sample was collected (1) or not (0). |
| 33 | Mortality | Whether the individual died while the GPS collar was active (1) or not (0). |
| 34 | MortDate | Inferred date of mortality in YYYY-MM-DD format. |
| 35 | MortCause | Inferred cause of death for individuals whose carcasses were inspected by project personnel. |
| 36 | OtherNotes | Contextual information. Field notes on body condition, reproductive status, social group size, capture details, and cases in which collars were immediately refurbished and redeployed on previously collared individuals; retrospective notes about collar malfunctions and causes of mortality; and other potentially relevant details. |

**C. Data anomalies:** Missing or non-applicable data are indicated by NA.

## 4. BUSHBUCK GPS COLLAR LOCATION DATA

### A. Data set file

**Identity:** GPS-COLLAR-LOCATION-RECORDS_BUSHBUCK.csv

**Size:** 55.4 MB

**Format:** CSV

**Contents:** Bushbuck location data. Includes animal identifier, date and time, and location of each GPS fix collected from bushbuck collared from 2014–2023 (*n* = 103 individuals, data from to June 2014 to May 2024). There are 656,828 lines of data, each containing the date, time, and location of one GPS fix. Measures of the accuracy of each GPS fix are provided where available.

### B. Variable information

| Column | Attribute | Definition |
|---|---|---|
| 1 | AnimalID | Unique individual ID (17 characters) including species code, year of collar deployment, sex (f, female; m, male), collar serial ID, and intra-annual collar deployment number (1 = first individual on which that GPS collar was deployed in that year; 2 = second individual on which that collar was deployed that year). |
| 2 | Species | Species common name |
| 3 | Timestamp | Calendar date and time of GPS fix in YYYY-MM-DD HH:MM:SS format, in local time (Central Africa Time, UTC+02:00). |
| 4 | Year | Year of GPS fix in YYYY format. |
| 5 | DayOfYear | Days from January 1st of the study year to the GPS-fix date. |
| 6–7 | Latitude, Longitude | Latitude and longitude of the GPS fix (WGS1984). |
| 8–9 | HDOP, DOP | Dilution of precision (DOP) metric for each GPS fix measured as either general spatial precision (DOP) or horizontal-specific precision (HDOP). |

**C. Data anomalies:** Missing data are indicated by NA.

## 5. ELAND GPS COLLAR LOCATION DATA

### A. Data set file

**Identity:** GPS-COLLAR-LOCATION-RECORDS_ELAND.csv

**Size:** 10.7 MB

**Format:** CSV

**Contents:** Eland location data. Includes animal identifier, date and time, and location of each GPS fix collected from eland collared from 2022–2023 (*n* = 10 individuals, data from June 2022 to May 2025). There are 131,173 lines of data, each containing the date, time, and location of one GPS fix. Measures of the accuracy of each GPS fix are provided where available.

### B. Variable information

| Column | Attribute | Definition |
|---|---|---|
| 1 | AnimalID | Unique individual ID (17 characters), including species code, year of collar deployment, sex (f, female; m, male), collar serial ID, and intra-annual collar deployment number (0 = not recorded; 1 = first individual on which that GPS collar was deployed in that year; 2 = second individual on which that collar was deployed that year). |
| 2 | Species | Species common name |
| 3 | Timestamp | Calendar date and time of GPS fix in YYYY-MM-DD HH:MM:SS format, in local time (Central Africa Time, UTC+02:00). |
| 4 | Year | Year of GPS fix in YYYY format. |
| 5 | DayOfYear | Days from January 1st of the study year to the GPS-fix date. |
| 6–7 | Latitude, Longitude | Latitude and longitude of the GPS fix (WGS1984). |
| 8–9 | HDOP, DOP | Dilution of precision (DOP) metric for each GPS fix measured as either general spatial precision (DOP) or horizontal-specific precision (HDOP, NA for all eland). |

**C. Data anomalies:** Missing data are indicated by NA.

## 6. ELEPHANT GPS COLLAR LOCATION DATA

### A. Data set file

**Identity:** GPS-COLLAR-LOCATION-RECORDS_ELEPHANT.csv

**Size:** 67 MB

**Format:** CSV

**Contents:** Elephant location data. Includes animal identifier, date and time, and location of each GPS fix collected from elephant collared from 2015–2018 (*n* = 18 individuals, data from December 2015 to August 2021). There are 839,947 lines of data, each containing the date, time, and location of one GPS fix. Measures of the accuracy of each GPS fix are provided where available.

### B. Variable information

| Column | Attribute | Definition |
|---|---|---|
| 1 | AnimalID | Unique individual ID (16 characters) including species code, year of collar deployment, sex (f, female; m, male), collar serial ID, and intra-annual collar deployment number (1 = first individual on which that GPS collar was deployed in that year; 2 = second individual on which that collar was deployed that year). |
| 2 | Species | Species common name |
| 3 | Timestamp | Calendar date and time of GPS fix in YYYY-MM-DD HH:MM:SS format, in local time (Central Africa Time, UTC+02:00). |
| 4 | Year | Year of GPS fix in YYYY format. |
| 5 | DayOfYear | Days from January 1st of the study year to the GPS-fix date. |
| 6–7 | Latitude, Longitude | Latitude and longitude of the GPS fix (WGS1984). |
| 8–9 | HDOP, DOP | Dilution of precision (DOP) metric for each GPS fix measured as either general spatial precision (DOP) or horizontal-specific precision (HDOP, NA for all elephant). |

**C. Data anomalies:** Missing data are indicated by NA.

## 7. KUDU GPS COLLAR LOCATION DATA

### A. Data set file

**Identity:** GPS-COLLAR-LOCATION-RECORDS_KUDU.csv

**Size:** 104.3 MB

**Format:** CSV

**Contents:** Kudu location data. Includes study animal identifier, date and time, and location of each GPS fix collected from kudu collared from 2014–2023 (*n* = 80 individuals, data from June 2014 to May 2025). There are 1,299,250 lines of data, each containing the date, time, and location of one GPS fix. Measures of the accuracy of each GPS fix are provided where available.

### B. Variable information

| Column | Attribute | Definition |
|---|---|---|
| 1 | AnimalID | Unique individual ID (17 characters) including species code, year of collar deployment, sex (f, female; m, male), collar serial ID, and intra-annual collar deployment number (1 = first individual on which that GPS collar was deployed in that year; 2 = second individual on which that collar was deployed that year). |
| 2 | Species | Species common name |
| 3 | Timestamp | Calendar date and time of GPS fix in YYYY-MM-DD HH:MM:SS format, in local time (Central Africa Time, UTC+02:00). |
| 4 | Year | Year of GPS fix in YYYY format. |
| 5 | DayOfYear | Days from January 1$^{st}$ of the study year to the GPS-fix date. |
| 6–7 | Latitude, Longitude | Latitude and longitude of the GPS fix (WGS1984). |
| 8–9 | HDOP, DOP | Dilution of precision (DOP) metric for each GPS fix measured as either general spatial precision (DOP) or horizontal-specific precision (HDOP). |

**C. Data anomalies:** Missing data are indicated by NA.

## 8. NYALA GPS COLLAR LOCATION DATA

### A. Data set file

**Identity:** GPS-COLLAR-LOCATION-RECORDS_NYALA.csv

**Size:** 36 MB

**Format:** CSV

**Contents:** Nyala location data. Includes study animal identifier, date and time, and location of each GPS fix collected from nyala collared from 2014–2022 (*n* = 37 individuals, data from June 2014 to November 2023). There are 444,809 lines of data, each containing the date, time, and location of one GPS fix. Measures of the accuracy of each GPS fix are provided where available.

### B. Variable information

| Column | Attribute | Definition |
|---|---|---|
| 1 | AnimalID | Unique individual ID (17 characters) including species code, year of collar deployment, sex (f, female; m, male), collar serial ID, and intra-annual collar deployment number (1 = first individual on which that GPS collar was deployed in that year; 2 = second individual on which that collar was deployed that year). |
| 2 | Species | Species common name |
| 3 | Timestamp | Calendar date and time of GPS fix in YYYY-MM-DD HH:MM:SS format, in local time (Central Africa Time, UTC+02:00). |
| 4 | Year | Year of GPS fix in YYYY format. |
| 5 | DayOfYear | Days from January 1st of the study year to the GPS-fix date. |
| 6–7 | Latitude, Longitude | Latitude and longitude of the GPS fix (WGS1984). |
| 8–9 | HDOP, DOP | Dilution of precision (DOP) metric for each GPS fix measured as either general spatial precision (DOP) or horizontal-specific precision (HDOP). |

**C. Data anomalies:** Missing data are indicated by NA.

## 9. WATERBUCK GPS COLLAR LOCATION DATA

### A. Data set file

**Identity:** GPS-COLLAR-LOCATION-RECORDS_WATERBUCK.csv

**Size:** 5.2 MB

**Format:** CSV

**Contents:** Waterbuck location data. Includes study animal identifier, date and time, and location of each GPS fix collected from waterbuck collared from 2015–2016 ($n$ = 22 individuals, data from August 2015 to September 2016). There are 61,744 lines of data, each containing the date, time, and location of one GPS fix.

### B. Variable information

| Column | Attribute | Definition |
|---|---|---|
| 1 | AnimalID | Unique individual ID (13–16 characters) including species code, year of collar deployment, sex (f, female; m, male), collar ID, and intra-annual collar deployment number (1 = first individual on which that GPS collar was deployed in that year; 2 = second individual on which that collar was deployed that year). |
| 2 | Species | Species common name |
| 3 | Timestamp | Calendar date and time of GPS fix in YYYY-MM-DD HH:MM:SS format, in local time (Central Africa Time, UTC+02:00). |
| 4 | Year | Year of GPS fix in YYYY format. |
| 5 | DayOfYear | Days from January 1$^{st}$ of the study year to the GPS-fix date. |
| 6–7 | Latitude, Longitude | Latitude and longitude of the GPS fix (WGS1984). |
| 8–9 | HDOP, DOP | Dilution of precision (DOP) metric for each GPS fix measured as either general spatial precision (DOP) or horizontal-specific precision (HDOP). Both measures NA for all waterbuck. |

**C. Data anomalies:** Missing data are indicated by NA.

## 10. ZEBRA GPS COLLAR LOCATION DATA

### A. Data set file

**Identity:** GPS-COLLAR-LOCATION-RECORDS_ZEBRA.csv

**Size:** 3.2 MB

**Format:** CSV

**Contents:** Zebra location data. Includes study animal identifier, date and time, and location of each GPS fix collected from zebras collared from 2022–2023 (*n* = 7 individuals, data from June 2022 to February 2024). There are 39,118 lines of data, each containing the date, time, and location of one GPS fix. Measures of the accuracy of each GPS fix are provided where available.

### B. Variable information

| Column | Attribute | Definition |
|---|---|---|
| 1 | AnimalID | Unique individual ID (17 characters) including species code, year of collar deployment, sex (f, female; m, male), collar serial ID, and intra-annual collar deployment number (1 = first individual on which that GPS collar was deployed in that year; 2 = second individual on which that collar was deployed that year). |
| 2 | Species | Species common name |
| 3 | Timestamp | Calendar date and time of GPS fix in YYYY-MM-DD HH:MM:SS format, in local time (Central Africa Time, UTC+02:00). |
| 4 | Year | Year of GPS fix in YYYY format. |
| 5 | DayOfYear | Days from January 1$^{st}$ of the study year to the GPS-fix date. |
| 6–7 | Latitude, Longitude | Latitude and longitude of the GPS fix (WGS1984). |
| 8–9 | HDOP, DOP | Dilution of precision (DOP) metric for each GPS fix measured as either general spatial precision (DOP) or horizontal-specific precision (HDOP, NA for all zebra). |

**C. Data anomalies:** Missing data are indicated by NA.

## 11. DIET COMPOSITION (RELATIVE-READ ABUNDANCE FROM RAREFIED DATA)

### A. Data set file

**Identity:** step8_final_dataset_rarefied_RRA.csv

**Size:** 5 MB

**Format:** CSV

**Contents:** Relative read abundance (RRA) of unique plant DNA sequences (molecular operational taxonomic units, mOTUs) detected in 3,785 fecal samples from 27 mammal species collected from August 2013 to July 2022. Includes identifiers, metadata, and diet composition for each fecal sample. RRA of each plant mOTU was calculated after rarefying all samples to 979 reads (*Class V.D.ii*). There are 3,785 rows (samples) and 532 columns (16 metadata columns and 516 mOTUs).

### B. Variable information

| Column | Attribute | Definition |
|---|---|---|
| 1 | Sample_ID | Sample identifier in the format: PNG_13_KOEL_001, where 'PNG' refers to Parque Nacional da Gorongosa; '13' is the last two digits of the sample collection year; 'KOEL' is the species code (see key at top of *Class IV*); and '001' is the chronological sample number for each species in a given year. |
| 2 | Sample_Sequencing_ID | Sample ID recorded in the field. Format is often similar to column 1 but varied in earlier years. |
| 3 | Species_Common_Name | The common name of the mammal species. |
| 4 | Species_Latin_Name | The Latin name of the mammal species. |
| 5 | Species_ID_Confirmed_Genetically | Categorical. 1 = sample not tested genetically; 2 = sample tested with inconclusive results; 3 = sample tested and species identity confirmed. |
| 6 | Sex | Female (F), male (M), or unknown (U) |
| 7 | Collection_Year | Year of collection in YYYY format. |
| 8 | Collection_Month | Calendar month of collection in MM format. |
| 9 | Collection_Date | Date of collection in YYYY-MM-DD format. |

| | | |
|---|---|---|
| 10 | Collection_Day_of_Year | Days from January 1st of the study year to the collection date. |
| 11 | Collection_Time_of_Day | Time of collection in 24-h HH:MM format. |
| 12 | Latitude | Latitude of sample collection location (WGS1984). |
| 13 | Longitude | Longitude of sample collection location (WGS1984). |
| 14 | Collection_Standard | Categorical standard of sample condition. 1 = not recorded; 2 = fresh dung but species of origin not seen in the vicinity; 3 = fresh dung and species of origin seen in the vicinity; 4 = defecation witnessed; 5 = sample collected directly from immobilized animal. |
| 15 | AnimalID | Unique identifier for GPS-collared individuals. Matches "AnimalID" variable in COLLAR-CAPTURE-FATE-RECORDS_2014-2023.csv. Values are only present for fecal samples collected from collared individuals; 'NA' indicates the sample was collected from an uncollared animal. |
| 16 | Mean_Read_Depth | Mean number of DNA sequence reads per sample across all PCR replicates for that sample. |
| 17–532 | Plant mOTUs | Columns are named using the following convention: [Lowest identifiable taxon]_[Plant family]_[DNA barcode sequence]:<br>Isoglossa floribunda_Acanthaceae_<br>atcctgttttctgaaaacaaaggtttcgag<br>Values are the proportion of reads (RRA) from the sample attributed to the mOTU and include integers (0 or 1) and decimals (0 > RRA > 1). Users can convert data into occurrence (presence–absence) format by simply assigning '1' to all nonzero values. |

**C. Data anomalies:** None.

## 12. DIET COMPOSITION (RAW, UNRAREFIED READ COUNTS)

### A. Data set file

**Identity:** step8_final_dataset_raw_reads.csv

**Size:** 4.7 MB

**Format:** CSV

**Contents:** Raw read counts of unique plant DNA sequences (molecular operational taxonomic units, mOTUs) detected in 3,785 fecal samples from 27 mammal species collected from August 2013 to July 2022. Includes identifiers, metadata, and diet composition for each fecal sample. Diet composition is reported as the mean number of reads assigned to each plant mOTU across all technical (PCR) replicates. As in data set *11*, there are 3,785 rows (samples) and 532 columns.

### B. Variable information

| Column | Attribute | Definition |
|---|---|---|
| 1 | Sample_ID | Sample identifier in the format: PNG_13_KOEL_001, where 'PNG' refers to Parque Nacional da Gorongosa; '13' is the last two digits of the sample collection year; 'KOEL' is the species code (see key at top of *Class IV*); and '001' is the chronological sample number for each species in a given year. |
| 2 | Sample_Sequencing_ID | Sample ID recorded in the field. Format is often similar to column 1 but varied in earlier years. |
| 3 | Species_Common_Name | The common name of the mammal species. |
| 4 | Species_Latin_Name | The Latin name of the mammal species. |
| 5 | Species_ID_Confirmed_Genetically | Categorical. 1 = sample not tested genetically; 2 = sample tested with inconclusive results; 3 = sample tested and species identity confirmed. |
| 6 | Sex | Female (F), male (M), or unknown (U) |
| 7 | Collection_Year | Year of collection in YYYY format. |
| 8 | Collection_Month | Calendar month of collection in MM format. |
| 9 | Collection_Date | Date of collection in YYYY-MM-DD format. |

| | | |
|---|---|---|
| 10 | Collection_Day_of_Year | Days from January 1st of the study year to the collection date. |
| 11 | Collection_Time_of_Day | Time of collection in 24-h HH:MM format. |
| 12 | Latitude | Latitude of sample collection location (WGS1984). |
| 13 | Longitude | Longitude of sample collection location (WGS1984). |
| 14 | Collection_Standard | Categorical standard of sample condition. 1 = not recorded; 2 = fresh dung but species of origin not seen in the vicinity; 3 = fresh dung and species of origin seen in the vicinity; 4 = defecation witnessed; 5 = sample collected directly from immobilized animal. |
| 15 | AnimalID | Unique identifier for GPS-collared individuals. Matches "AnimalID" variable in COLLAR-CAPTURE-FATE-RECORDS_2014-2023.csv. Values are only present for fecal samples collected from collared individuals; 'NA' indicates that the sample was collected from an uncollared animal. |
| 16 | Mean_Read_Depth | Mean number of DNA sequence reads per sample across all PCR replicates for that sample. |
| 17–532 | Plant mOTUs | Columns are named using the following convention: [Lowest identifiable taxon]_[Plant family]_[DNA barcode sequence]:<br>Isoglossa floribunda_Acanthaceae_<br>atcctgttttctgaaaacaaaggtttcgag<br>Values are the mean number of reads across PCR replicates attributed to the mOTU and include both integers and decimals. |

**C. Data anomalies:** None.

## 13. FECAL SAMPLE METADATA

### A. Data set file

**Identity:** step8_final_metadata.csv

**Size:** 515 KB

**Format:** CSV

**Contents:** Identifiers and metadata for 3,785 fecal samples from 27 mammal species collected from August 2013 to July 2022. There are 3,785 rows (samples) and 17 metadata columns.

### B. Variable information

| Column | Attribute | Definition |
|---|---|---|
| 1 | Sample_Sequencing_ID | Sample ID recorded in the field. Format is often similar to column 16 but varied in earlier years. |
| 2 | Original_Species_ID | Common name of the mammal species recorded in the field. In some cases, this differs from the final common name (column 12) when species identity was revised based on analysis of mammal DNA. |
| 3 | Sex | Female (F), male (M), or unknown (U) |
| 4 | Collection_Year | Year of collection in YYYY format. |
| 5 | Collection_Month | Calendar month of collection in MM format. |
| 6 | Collection_Date | Date of collection in YYYY-MM-DD format. |
| 7 | Collection_Day_of_Year | Days from January 1st of the study year to the collection date. |
| 8 | Collection_Time_of_Day | Time of collection in 24-h HH:MM format. |
| 9 | Latitude | Latitude of sample collection location (WGS1984). |
| 10 | Longitude | Longitude of sample collection location (WGS1984). |
| 11 | Collection_Standard | Categorical standard of sample condition. 1 = not recorded; 2 = fresh dung but species of origin not seen in the vicinity; 3 = fresh dung and species of origin seen in the vicinity; 4 = defecation witnessed; 5 = sample collected directly from immobilized animal. |

| | | |
|---|---|---|
| 12 | Species_Common_Name | The common name of the mammal species. |
| 13 | Species_Latin_Name | The Latin name of the mammal species. |
| 14 | AnimalID | Unique identifier for GPS-collared individuals. Matches "AnimalID" variable in COLLAR-CAPTURE-FATE-RECORDS_2014-2023.csv. Values are only present for fecal samples collected from collared individuals; 'NA' indicates that the sample was collected from an uncollared animal. |
| 15 | Species_ID_Confirmed_Genetically | Categorical. 1 = sample not tested genetically; 2 = sample tested with inconclusive results; 3 = sample tested and species identity confirmed. |
| 16 | Sample_ID | Sample identifier in the format: PNG_13_KOEL_001, where 'PNG' refers to Parque Nacional da Gorongosa; '13' is the last two digits of the sample collection year; 'KOEL' is the species code (see key at top of *Class IV*); and '001' is the chronological sample number for each species in a given year. |
| 17 | Mean_Read_Depth | Mean number of DNA sequence reads per sample across all PCR replicates for that sample. |

**C. Data anomalies:** None.

## 14. TAXONOMIC INFORMATION FOR PLANT DNA BARCODES

### A. Data set file

**Identity:** step8_final_mOTU_taxonomy.csv

**Size:** 142 KB

**Format:** CSV

**Contents:** Taxonomic identifiers and matching between fecal DNA and reference DNA libraries for each of the 516 plant molecular operational taxonomic units (mOTUs) retrieved from 3,785 fecal samples collected from 2013–2022. There are 516 rows (plant mOTUs) and 17 columns.

### B. Variable information

| Column | Attribute | Definition |
|---|---|---|
| 1 | id | Plant DNA sequence identifier in the format seq_[1:516] |
| 2 | count | Sum of unrarefied read counts (data set *12*) for each mOTU across all fecal samples. |
| 3 | sequence | The DNA barcode sequence associated with each plant mOTU, with lengths ranging from 18–137 bp (median 51 bp, IQR 46–56 bp). |
| 4 | db | Categorical; indicates whether sequence was best matched by either of two reference DNA databases: (i) a local library of plant DNA from reference specimens collected in Gorongosa (data set *20*) or a (ii) global library of plant DNA compiled from the European Molecular Biology Laboratory (EMBL; release 143). 'localDB' indicates that the sequence best matched a specimen collected in Gorongosa; 'globalDB' indicates a better match to a sequence in the EMBL database. |
| 5 | bid | Proportional match between the sequence and its best match in the database used for identification (column 4). 1 = perfect match (identical sequences). 0.98 = 98% match (e.g., a difference of 1 bp in a median-length sequence of 50 bp). |
| 6 | taxid | The National Center for Biotechnology Information (NCBI) taxonomic identifier associated with the reference sequence best matching the focal sequence. |
| 7 | sci_name | The most resolved taxonomic name possible based solely on information in the DNA reference library. |

| | | |
|---|---|---|
| 8 | bmatch | Accession number for the reference sequence that provided the best match to each mOTU. |
| 9 | rank | Taxonomic rank associated with the name in column 7. |
| 10 | order_name | Botanical order of the mOTU based on the best match in the reference library (column 7). If 'NA', the mOTU could not be uniquely identified to order (*n* = 5 of 516 mOTUs, 1.0%). |
| 11 | family_name | Botanical family of the mOTU based on the best match in the reference library (column 7). If 'NA', the mOTU could not be uniquely identified to family (*n* = 17 of 516 mOTUs, 3.3%). |
| 12 | genus_name | Botanical genus of the mOTU based on the best match in the reference library. If 'NA', mOTU could not be uniquely identified to genus (*n* = 167 of 516 mOTUs, 32.4%). |
| 13 | species_name | Latin binomial of the mOTU based on the best match in the reference library. If 'NA', the mOTU could not be uniquely identified to species (n = 323 of 516 mOTUs, 62.6%). |
| 14 | Resolved | 'Yes' or 'No'. If 'Yes', the mOTU was included in the post hoc taxonomic assignment process that refined the identification using botanical references for Mozambique generally and Gorongosa specifically (see *Class V.D.ii*). |
| 15 | Resolved_Species List | List of species names, separated by semicolons, that plausibly match the mOTU based on the decision rules in *Class V.D.ii* (see column 9 in data set *15*). Leading asterisks ('*') signify that the listed species are not currently known to occur in Mozambique or PNG. 'NA' indicates that the mOTU was not included in the post hoc refinement process. |
| 16 | Resolved_FinalID | Simplified mOTU identity based on 'Resolved_SpeciesList', consisting of one or two species names, separated by hyphens, if < 3 species (e.g., Urochloa trichopus-Eriochloa barbata), or else the lowest wholly inclusive taxonomic grouping (e.g., Caesalpinioideae, PACMAD clade, Meliaceae). 'NA' if the mOTU was not included in the post hoc refinement process. |
| 17 | CombinedID | Working ID for each mOTU in the format [Most precise taxonomic name]_[plant family]_[barcode sequence], e.g.:<br>Tephrosia.pumila_Fabaceae_<br>atcctattttccaaaataagttcgaataaaaaaag<br>For mOTUs included in the post-hoc refinement process, the first taxonomic name is the mOTU’s 'Resolved_FinalID'; for mOTUs not included in the post-hoc process, the first value is the mOTU’s 'sci_name'. This variable corresponds to the non-metadata column names in data sets *11* and *12* above. |

**C. Data anomalies:** None.

## 15. POST-HOC INVESTIGATION OF TAXONOMIC INFORMATION FOR 145 OF THE MOST COMMON PLANT DNA BARCODES

### A. Data set file

**Identity:** step7_resolved_mOTUs.csv

**Size:** 54 KB

**Format:** CSV

**Contents:** For the 145 most common mOTUs detected across all fecal samples, which together accounted for >90% of DNA reads in data sets *11* and *12*, this table reports their DNA barcode sequence, best match to the local DNA reference library, the results of querying the National Center for Biotechnology Infrastructure (NCBI) megablast algorithm for each sequence, and the filtering of those results based on credible botanical compilations for Mozambique and Gorongosa. By combining this bioinformatic and biogeographic information, we conducted a systematic post-hoc refinement of the taxonomic identity for these barcodes, as reported in the 'FinalID' column of this file and the 'CombinedID' column of data set *14* (see *Class V.D.ii* below for sources and methodology). The file contains 145 rows (mOTUs) and 11 columns.

### B. Variable information

| Column | Attribute | Definition |
|---|---|---|
| 1 | Barcode | DNA barcode sequence associated with the plant mOTU. |
| 2 | PercentMatch_PNG | Percentage match between the mOTU and its best match in the local reference database. 100 = identical sequences. |
| 3 | SpeciesList_PNG | All species in the local DNA reference database that best match the mOTU. Multiple species names are separated by semi-colons. |
| 4 | ID_PNG | The most specific taxonomic name that could be assigned to the mOTU based solely on records in the Gorongosa DNA reference library. |
| 5 | QueryCov_BLAST_percent | Output from the BLAST algorithm; percentage of the mOTU sequence that is aligned to the best-matching reference sequence(s). |
| 6 | PerIdent_BLAST | Output from the BLAST algorithm; the percent of nucleotides in the focal DNA barcode that are identical in the alignment between the mOTU and the best-matching reference sequence(s). |

| | | |
|---|---|---|
| 7 | Matches_in_Moz | List of matching species from the BLAST query that are known to occur in Mozambique, based on Kew Garden's Plants of the World Online database (https://powo.science.kew.org /). Species names are separated by semicolons. |
| 8 | Matches_in_PNG | List of matching species from the BLAST query known to occur in PNG, based on the Flora of Mozambique (https://www.mozambiqueflora.com/) and records from PNG's own botanical inventory. Species names are separated by semicolons. |
| 9 | FinalID | List of species (Latin names) deemed most likely to match the mOTU, based on the decision rules outlined in *Class V.D.ii*. Entries are separated by semicolons. Leading asterisks ('*') signify that the listed species are not currently known to occur in Mozambique or PNG. |
| 10 | TidyFinalID | Simplest characterization of 'FinalID', comprising concatenated species names (if fewer than three) or the lowest inclusive taxonomic grouping (if three or more species). This column is equivalent to the Resolved_FinalID (column 16) in data set *14*. |
| 11 | Notes | Notes about the assignment, e.g., to explain the exclusion of a plausible matching species (usually if that species uniquely matched a different sequence) or to clarify the reasons for asterisks in 'FinalID' (signifying that the putatively matching species in that column are not currently known to occur in Mozambique or PNG). |

**C. Data anomalies:** None.

## 16. MAPPING THE REFINED TAXONOMIC IDENTIFICATIONS TO THOSE USED IN PREVIOUS WORK

### A. Data set file

**Identity:** mOTU_matching_Potter2022.csv

**Size:** 90 KB

**Format:** CSV

**Contents:** A correspondence matrix linking our refined mOTU IDs to previous work from PNG. Potter et al. (2022) investigated mechanisms of dietary resource partitioning by large herbivores in PNG by measuring 27 functional traits for 204 common plant species and analyzing diet composition in 286 fecal samples from mid-2018. To facilitate use of those trait data with our data sets *11* and *12* (which include the samples used by Potter et al.), we provide a table connecting the mOTU assignments in data set *14* to Potter et al.'s 'trait operational taxonomic units' (TOTUs: mOTUs that had trait data for at least one of the species). Most of the 516 mOTUs described here were not assigned to TOTUs by Potter et al. ('NA' in column 4), either because they were not detected or because they lacked trait data. Of the 96 mOTUs in our data that were mapped to TOTUs by Potter et al., the taxonomy in Potter et al. (2022) is largely compatible with our refined identifications. For 44 of them, the identifications are identical [homotypic synonyms: *Berchemia*–*Phyllogeiton* (*discolor*), *Eichhornia*–*Pontederia* (*crassipes*), *Heliotropum*–*Euploca* (*ovalifolia*), *Pteleopsis*–*Terminalia* (*myrtifolia*)]. In all but 2 of the rest, the ID used by Potter et al. is a subset of the 'CombinedID' in data set *14*, or vice versa [synonyms: *Acacia*–*Senegalia* (spp.), *Acacia*–*Vachellia* (spp.), *Urochloa mosambicensis*–*U. trichopus*, *Digitaria swazilandensis*–*D. didactyla*, *Panicum*–*Megathyrsus* (*maximus*)]. Conflicts: seq_17 in data set *14*, identified here as *Cenchrus purpureus*, was labeled as *Setaria incrassata* (a related grass; subfamiliy Panicoideae) in Potter et al. (2022), apparently due to a specimen misidentification in the local DNA reference database (JG-16-P03_C3, identified as "*Setaria* sp."); likewise, seq_145 in data set *14*, identified here as *Sesbania bispinosa*, was labeled by Potter et al. as a congener, *Sesbania sesban* (seq_78 in data set *14*), based on a reference specimen identified only to genus. Future analyses should re-evaluate the trait:mOTU correspondence in light of the refined identities here and Appendix 3 of Potter et al. (2022). The file contains 516 rows and 6 columns.

### B. Variable information

| Column | Attribute | Definition |
|---|---|---|
| 1 | Sequence | DNA barcode sequence associated with the plant mOTU. |
| 2 | CombinedID | Current ID for each mOTU in the format [Most precise taxonomic name]_[plant family]_[barcode sequence] (see column 17 in data set *14*). |

| | | |
|---|---|---|
| 3 | Resolved_SpeciesList | List of species names that plausibly match the mOTU (see column 15 in data set *14*). |
| 4 | PotterSpeciesID | mOTU taxonomic identifier used by Potter et al. (2022). |
| 5 | PotterTOTU | 'Trait Operational Taxonomic Unit', identifier used by Potter et al. (2022) to coarse-grain species' trait data to the level of mOTUs by averaging trait values within species and across species in multispecies mOTUs. |
| 6 | PotterSpeciesIDHarmonized | All original PotterSpeciesID values updated to match the botanical nomenclature used here (Plants of the World Online, Kew Botanical Gardens). |

**C. Data anomalies:** None.

## 17. PRIMER–TEMPLATE ALIGNMENTS FOR DNA DIETARY METABARCODING

### A. Data set file

**Identity:** primer_template_alignments.csv

**Size:** 91 KB

**Format:** CSV

**Contents:** For each mOTU in data sets *11*, *12*, and *14*, we report its alignment with the P6 *trn*L (UAA) primers (*g*, *h*) at the binding sites. Each row in the table reports, for one mOTU, the match to each primer, the number of mismatches, and position-by-position matches for each primer. Position-by-position column names are numbered by primer and position, beginning at the 5' end (e.g., 'F1' for the first base from the 5' end of the forward primer); these data are binary, where '0' represents a match between primer and template at that position and '1' represents mismatch. Two mOTUs had one mismatch with the forward primer. One mismatch was in the middle of the primer; the other was 4 bp from the 3' end. Eight mOTUs had one mismatch to the reverse primer. All mismatches with the reverse primer occurred in the middle of the primer; six of which were at position 16. No mOTUs had more than one mismatch per primer, but one mOTU had one mismatch on each primer. The file contains 516 rows and 44 columns.

### B. Variable information

| Column | Attribute | Definition |
|---|---|---|
| 1 | Sequence | DNA barcode sequence associated with the plant mOTU. |
| 2 | ForwardMatch | The region flanking the barcode region matched to the forward primer (5′-GGGCAATCCTGAGCCAA-3′). |
| 3 | ReverseMatch | Region flanking the barcode region matched to the reverse primer (5′- CCATTGAGTCTCTGCACCTATC -3′). |
| 4 | FowardPrimerMismatches | Mismatches between forward primer and ForwardMatch. |
| 5 | ReversePrimerMismatches | Mismatches between reverse primer and ReverseMatch. |
| 6–22 | ForwardPrimerAlignments (F1–F17) | Each column represents one position on the primer, from the 5' end. Binary: '0' indicates a match at this position between the template and primer; '1' indicates a mismatch. |
| 23-44 | ReversePrimerAlignments (R1–R22) | Each column represents one position on the primer, from 5' end. Values are binary: '0' indicates a match at this position between the template and primer; '1' represents a mismatch. |

**C. Data anomalies:** None.

## 18. RAW DNA METABARCODING DATA: DIET COMPOSITION

### A. Data set files

| File Name | Sampling Year | Sampling Season | Size |
|---|---|---|---|
| png13_jul_lib1_assigned.fastq.gz | 2013 | Early dry | 60 MB |
| png13_jul_lib2_assigned.fastq.gz | 2013 | Early dry | 23 MB |
| png14_jul_assigned.fastq.gz | 2014 | Early dry | 258 MB |
| png15_jul_lib1_assembled_assigned.fastq.gz | 2015 | Early dry | 3,130 MB |
| png15_jul_lib2_assembled_assigned.fastq.gz | 2015 | Early dry | 2,800 MB |
| png15_jul_lib3_assembled_assigned.fastq.gz | 2015 | Early dry | 2,270 MB |
| png15_jul_lib4_assembled_assigned.fastq.gz | 2015 | Early dry | 3,250 MB |
| png15_jul_lib5_assembled_assigned.fastq.gz | 2015 | Early dry | 4,270 MB |
| png15_jul_part2_assigned.fastq.gz | 2015 | Early dry | 636 MB |
| png16_jul_lib1_assembled_assigned.fastq.gz | 2016 | Early dry | 6,050 MB |
| png16_jul_lib2_assembled_assigned.fastq.gz | 2016 | Early dry | 4,170 MB |
| png16_jul_lib3_assembled_assigned.fastq.gz | 2016 | Early dry | 3,310 MB |
| png17_jul_assembled_assigned.fastq.gz | 2017 | Early dry | 3,630 MB |
| png17_julRedo_nov_assembled_assigned.fastq.gz | 2017 | Dry | 2,260 MB |
| png18_apr_assembled_assigned.fastq.gz | 2018 | Late wet | 2,010 MB |
| png18_jul_lib1_assembled_assigned.fastq.gz | 2018 | Early dry | 5,160 MB |
| png18_jul_lib2_assembled_assigned.fastq.gz | 2018 | Early dry | 5,170 MB |
| png18_jul_lib3_assembled_assigned.fastq.gz | 2018 | Early dry | 5,200 MB |
| png18_nov_assembled_assigned.fastq.gz | 2018 | Late dry | 2,270 MB |
| png19_apr_assembled_assigned.fastq.gz | 2019 | Late wet | 449 MB |
| png19_jul_lib1_assembled_assigned.fastq.gz | 2019 | Early dry | 3,680 MB |

| | | | |
|---|---|---|---|
| png19_jul_lib2_assembled_assigned.fastq.gz | 2019 | Early dry | 3,310 MB |
| png19_jul_lib3_assembled_assigned.fastq.gz | 2019 | Early dry | 3,930 MB |
| png19_nov_assembled_assigned.fastq.gz | 2019 | Late dry | 6,520 MB |
| png20_apr_assembled_assigned.fastq.gz | 2020 | Late wet | 2,140 MB |
| png21_jul_lib1_assembled_assigned.fastq.gz | 2021 | Early dry | 479 MB |
| png21_jul_lib2_assembled_assigned.fastq.gz | 2021 | Early dry | 622 MB |
| png21_jul_lib3_assembled_assigned.fastq.gz | 2021 | Early dry | 627 MB |
| png21_22_lib1_assembled_assigned.fastq.gz | 2021-2022 | All | 444 MB |
| png21_22_lib2_assembled_assigned.fastq.gz | 2021-2022 | All | 459 MB |
| png21_22_lib3_assembled_assigned.fastq.gz | 2021-2022 | All | 479 MB |

**Format:** FASTQ

**Contents:** Unfiltered DNA sequences retrieved from herbivore fecal samples when amplifying DNA from the P6 loop of the *trn*L intron in the chloroplast genome. See *Class II.B.3.a–b.iii* above for laboratory procedures. These data were preprocessed using the OBITools software v1.2.11 (Boyer et al. 2016) to (1) align paired-end sequences, retaining sequences with high alignment quality, and (2) assign retained sequences to their fecal sample of origin. See *Class V.D.ii* below for complete description of these bioinformatic steps. Sequence records include the unique sequencing identifier, semicolon-separated output attributes from *illuminapairedend* and *ngsfilter* processing, the DNA sequence, and the Phred +33 encoded quality scores for each base.

**B. Variable information**

| **Attribute** | **Definition** |
|---|---|
| sample | Sequencing identifier (Sample_Sequencing_ID in data sets *11–13*). |
| experiment | Name of the sequencing library that the sequence and sample belong to. |
| seq_length | Length of DNA barcode. |
| seq_length_ori | Length of sequence before primer and tag removal. |
| forward_score | Alignment score of the 5' primer against the sequence. |
| forward_tag | Sample-specific tag sequence appended in 5' of the forward primer, used to assign sequences to samples. Synonymous with 'direct_tag' and 'Ftag'. |

| | |
|---|---|
| forward_primer | Forward primer used to obtain the sequence. |
| forward_match | Forward primer match used to identify the barcode. |
| reverse_tag | Sample-specific tag sequence appended in 5' of the reverse primer, used to assign sequences to samples. Synonymous with 'Rtag'. |
| reverse_score | Alignment score of the 3' primer against the sequence. |
| reverse_match | Reverse primer match used to identify the barcode. |
| reverse_primer | Reverse primer used to obtain the sequence. |
| status | Whether the amplicon has been sequenced in its entirety; 'full' if so, 'partial' if not. |
| direction | Whether the primers were identified on the forward ('forward') or reverse ('reverse') strand. |
| ali_length | Length of alignment between the two paired-end reads. |
| score | Quality score computed based on the alignment of the two paired-end reads. |
| score_norm | Quality score based on the alignment score divided by the alignment length. |
| mode | Whether the reported sequence is the concatenation of the two reads ('joined') or the consensus alignment of them ('aligned'). |
| seq_ab_match | Number of matches in the aligned part of the sequence. |
| sminL | Minimum score above which 'left' alignment is discarded. |
| sminR | Minimum score above which 'right' alignment is discarded. |
| seq_b_mismatch | Number of mismatches between the second read and the consensus sequence in the aligned part. |
| seq_a_mismatch | Number of mismatches between the first read and the consensus sequence in the aligned part. |
| seq_b_insertion | Number of insertions between the second read and the consensus sequence in the aligned part. |
| seq_a_insertion | Number of insertions between the first read and the consensus sequence in the aligned part. |

| | |
|---|---|
| seq_b_deletion | Number of deletions between the second read and the consensus sequence in the aligned part. |
| seq_a_deletion | Number of deletions between the first read and the consensus sequence in the aligned part. |
| seq_b_single | Number of nucleotides of the second read that belong to the consensus sequence and were not aligned with the first read. |
| seq_a_single | Number of nucleotides of the first read that belong to the consensus sequence and were not aligned with the second read. |
| head_quality | Average base quality score of the first 10 nucleotides of the barcode. |
| mid_quality | Average base quality score of the barcode except for the first and last 10 nucleotides. |
| tail_quality | Average base quality score of the last 10 nucleotides of the barcode. |
| avg_quality | Average quality across the entire sequence. |

**C. Data anomalies:** None.

## 19. RAW DNA METABARCODING DATA: SAMPLE IDENTIFICATION

### A. Data set files

| File Name | Sampling Years | Size |
|---|---|---|
| sample_id_test_lib1_assembled_assigned.fastq.gz | 2016–2020 | 2,330 MB |
| sample_id_test_lib2_assembled_assigned.fastq.gz | 2016–2020 | 1,930 MB |
| sample_id_test_2022_lib1_assembled_assigned.fastq.gz | 2021–2022 | 309 MB |
| sample_id_test_2023_lib1_assembled_assigned.fastq.gz | 2021–2022 | 144 MB |
| sample_id_test_2023_lib2_assembled_assigned.fastq.gz | 2021–2022 | 105 MB |

**Format:** FASTQ

**Contents:** Unfiltered sequences retrieved from mammal fecal samples when amplifying DNA from the mitochondrial 16S rRNA gene. See *Class II.B.3.a–b.iii* above for laboratory procedures. These data were preprocessed using the OBITools software v1.2.11 (Boyer et al. 2016) to (1) align paired-end sequences, retaining sequences with high alignment quality, and (2) assign retained sequences to their fecal sample of origin. See *Class V.D.ii* below for complete description of these bioinformatic steps. Sequence records include unique sequencing identifier, semicolon-separated output attributes from *illuminapairedend* and *ngsfilter* processing, the DNA sequence, and the Phred +33 encoded quality scores for each base.

### B. Variable information

| Attribute | Definition |
|---|---|
| sample | Sequencing identifier (Sample_Sequencing_ID in data sets *11–13*). |
| experiment | Name of sequencing library for the sequence and sample. |
| seq_length | Length of DNA barcode. |
| seq_length_ori | Length of sequence before primer and tag removal. |
| forward_score | Alignment score of the 5' primer against the sequence. |
| forward_tag | Sample-specific tag sequence appended in 5' of the forward primer. Synonymous with 'direct_tag' and 'Ftag'. |

| | |
|---|---|
| forward_primer | Forward primer used to obtain the sequence. |
| forward_match | Forward primer match used to identify the barcode. |
| reverse_tag | Sample-specific tag sequence appended in 5' of the reverse primer. Synonymous with 'Rtag'. |
| reverse_score | Alignment score of the 3' primer against the sequence. |
| reverse_match | Reverse primer match used to identify the barcode. |
| reverse_primer | Reverse primer used to obtain the sequence. |
| status | Either 'full' if the amplicon has been sequenced in its entirety or 'partial' if not. |
| direction | Either 'forward' or 'reverse'. Indicates if the primers were identified on the forward or reverse strand. |
| ali_length | Length of the alignment between the two paired-end reads. |
| score | A quality score computed based on the alignment of the two paired-end reads. |
| score_norm | A quality score based on the alignment score divided by the alignment length. |
| mode | Either 'alignment' or 'joined'. Indicates whether the reported sequence is the concatenation of the two reads ('joined') or the consensus alignment of them ('aligned'). |
| seq_ab_match | Number of matches in the aligned part of the sequence. |
| sminL | Minimum score above which 'left' alignment is discarded. |
| sminR | Minimum score above which 'right' alignment is discarded. |
| seq_b_mismatch | Number of mismatches between the second read and the consensus sequence in the aligned part. |
| seq_a_mismatch | Number of mismatches between the first read and the consensus sequence in the aligned part. |
| seq_b_insertion | Number of insertions between the second read and the consensus sequence in the aligned part. |

| | |
|---|---|
| seq_a_insertion | Number of insertions between the first read and the consensus sequence in the aligned part. |
| seq_b_deletion | Number of deletions between the second read and the consensus sequence in the aligned part. |
| seq_a_deletion | Number of deletions between the first read and the consensus sequence in the aligned part. |
| seq_b_single | Number of nucleotides of the second read that belong to the consensus sequence and were not aligned with the first read. |
| seq_a_single | Number of nucleotides of the first read that belong to the consensus sequence and were not aligned with second read. |
| head_quality | Average base quality score of the first 10 nucleotides of the barcode. |
| mid_quality | Average base quality score the barcode except the first and last 10 nucleotides. |
| tail_quality | Average base quality score of the last 10 nucleotides of the barcode. |
| avg_quality | Average quality across the entire sequence. |

**C. Data anomalies:** None.

## 20. FILTERING SCRIPTS

### A. Data set files

| Directory Name | Function |
|---|---|
| GPS-collar-location-data-processing.R | Script to clean raw GPS movement data into deposited versions. Raw data are available from R.A.L. |
| filtering_prerequisites.txt | Instructions on initial setup and requirements for users wishing to refilter raw DNA metabarcoding data (data sets *18* and *19*). |
| bioinformatics_step1/ | Directory containing scripts to filter raw sequence data using OBITools software. |
| bioinformatics_step2/ | Directory containing scripts to filter outputs from step 1 using R Statistical Software. |
| ngsfilter_files/ | File reporting primers, dual-index tags, and plate location for each sample in each raw data set. |
| metadata_files/ | Original metadata for each sample, generally one file per year. These files are called in the filtering scripts but modified within those scripts. Users should refer to data set *13* for metadata when using the filtered diet data sets (data sets *11* & *12*). |
| additional_files_scripts/ | Scripts and data files called during bioinformatics steps 1 and 2, along with additional scripts (filenames beginning with 'step…') and data files to recreate the final data sets ('step8'). |

**Format:** Indicated by file-name extension: '.txt' are plain text files; '.r' or '.R' are in R Statistical Software language; '.RData' are data files outputted by R; '.fasta' are FASTA files containing DNA sequence data; '.xlsx' are tables formatted for Microsoft Excel; '.csv' are comma-separated data tables; '.sh' are coding scripts written for the BASH program; '.ngsfilter' are tab-separated tables used by the *ngsfilter* pre-processing function in OBITools.

**Contents:** All code and additional files required for the programming environment to filter raw DNA sequences (data sets *18* and *19*) into their final forms (data sets *11–14* above). Code is commented. Note: these files should only be used to refilter the raw data provided on Dryad (DOI: 10.5061/dryad.m905qfv85). The file structure enables all bioinformatic steps for cleaning the diet metabarcoding data, provided the user follows the steps in the "filtering_prerequisites.txt" file.

**Class V. Supplemental descriptors**

**A. Data acquisition**

**1. Data forms or acquisition methods:** Raw sequencing and GPS data were downloaded directly from the institutional and manufacturer platforms where data were stored. Metadata for each fecal sample or capture event were digitized and verified by hand.

**2. Location of completed data forms:** Metadata were recorded on paper in the field and promptly digitized. Animal capture data sheets are stored in R.A.L.'s laboratory at the University of Idaho. GPS-collar data are stored on OneDrive at the University of Idaho. All fecal samples and DNA extracts are stored in R.M.P.'s laboratory at Princeton University, along with laboratory notebooks and logs. DNA metabarcoding data are archived on long-term storage platforms at Princeton University and the University of California, Merced. See also *Class III.B.1* and *Class V.E.2*.

**3. Data entry verification procedures:** Manual data-entry was cross-checked against the physical datasheets. Instrument-generated data (GPS locations, sequence data) were carefully filtered and scrutinized for plausibility and consistency (see also *Class II.B.3* and *Class V.B* and *V.D*).

**B. Quality assurance/quality control procedures:**

**i. Rainfall data:** From 2015 onwards, rainfall was measured by an automatic weather station, with a manual rain gauge used for validation. Rainfall data from previous years were recorded using only a manual gauge, with several anomalies noted in *Class IV*, data sets *1* and *2*.

**ii. GPS collar data:** GPS data are raw. In general, the only filtering steps we performed were to (a) trim fixes to those collected after deployment and before the collar was dropped from each individual, and (b) remove data points that lacked a timestamp (e.g., date but no time, or neither date nor time), which occurred at the end of a collar's battery life. Waterbuck were the exception to this approach; following Becker et al. (2021), we rarefied fixes to be ≥ 5 min apart. Where possible, we report collar-derived dilution of precision (DOP; horizontal dilution of precision, HDOP) alongside GPS fixes in the data files (see *Class IV* data sets *4–10*).

**iii. Fecal DNA metabarcoding:** At the time of collection, we reduced the chance of environmental contamination by sampling from clean upwards-facing dung pellets. During laboratory analyses, we used negative controls during both DNA extraction and PCR to assess cross-contamination and identify contaminants. We left 12 wells per 96-well plate empty during PCR to assess the potential for tag jumps. Median sequencing error rates on Illumina platforms are estimated to be ≈ 0.1% for HiSeq 2500 and NovaSeq 6000 platforms and 0.5% for MiSeq platforms (Stoler and Nekrutenko 2021). Last, we used a systematic series of steps to filter sequencing data and refine taxonomic assignments in final data sets (see *Class V.D*). Collectively, these steps, distributed throughout the analytical pipeline, reduce the risk of error in the DNA metabarcoding data.

**C. Related materials:** NA

**D. Computer programs and data-processing algorithms:**
**i. GPS collar data:** Not applicable (see *Class V.B*).

**ii. Fecal DNA metabarcoding:** To analyze herbivore diet composition (*Class IV.11–15*), we applied the following steps to produce the final filtered data sets. Sequencing results were filtered using the OBITools pipeline (v.1.2.11; Boyer et al. 2016). Preprocessing included demultiplexing sequencing runs into libraries and assembling reads, when necessary, followed by demultiplexing sequencing libraries to individual fecal samples. After 2015, sequence data were demultiplexed automatically by Princeton University's Genomics Core Facility; in previous years, we inputted the Illumina index tags to the *obigrep* program with argument *illumina_index* to demultiplex runs. Single-end reads from 2013, 2014, and some of 2015 did not require assembly; paired-end reads (from 2015 onwards) were assembled with the *illuminapairedend* command (arguments: *--sanger --score-min=40*). Sequences were then assigned to samples (*ngsfilter* command; arguments: *-e 2*).

Primary bioinformatic filtering involved several steps to remove low-quality sequences, aggregate identical sequences, assign sequences to taxa, and assess which sequences might represent PCR or sequencing errors. Identical sequences were merged, and unique sequence labels were assigned using *obiuniq -m sample* and *obiannotate --set-identifier='"seq_%03d" % counter'*. Low-quality sequences—indicated by unexpected barcode length (< 8 bp or >180 bp; Taberlet et al. 2018),

ambiguous nucleotides, or assembled paired-end reads with a low alignment score (mode ≠ 'alignment')—were discarded. Sequences with extremely low abundance across an entire data set ($n < 10$ reads) were also discarded. The remaining sequences were considered mOTUs. Taxonomic identifiers were assigned to mOTUs using an extensive but non-exhaustive reference database for the P6 loop of the chloroplast *trn*L (UAA) intron, based on DNA extracted from plant specimens collected in PNG (Pansu et al. 2019), along with a global reference database compiled using *in silico* PCR and the European Molecular Biology Laboratory nucleotide sequence database (release 143). Any sequences not classified as part of Embryophyta (NCBI taxid: 3193) were discarded. Finally, mOTUs were compared to each other to identify variants of abundant sequences in the data set (*obiclean -d 1 -s merged sample -r 0.25*). Variant sequences—those classified as "internal" to other sequences more often than they were classified as "head" or "singleton" sequences—were removed unless they perfectly matched a local-reference-database sequence.

Secondary bioinformatic filtering involved refinement of taxonomic assignment, identification and removal of putative contaminants and chimeras, removal of PCR replicates with low sequencing depth, and assessment of PCR replicability. All mOTUs were preferentially assigned to the best-matching taxon in the local reference database if the best-match between reference and mOTU was > 98%; if not, assignment defaulted to the global reference library. mOTUs were discarded as putative contaminants or chimeras, respectively, if they were most abundant in negative controls or if their best match to a reference-database sequence was < 80%. We discarded PCR replicates with read counts of these erroneous sequences that were above the $95^{th}$ quantile. We also discarded PCR replicates if sequencing depth was < 1000 reads or fell below the $5^{th}$ quantile of read depth for the sequencing run, whichever was larger. The compositional consistency of PCR replicates was quantified as the Bray-Curtis dissimilarity among sample replicates, calculated with the *vegan* package (v2.5.6; Oksanen et al. 2013) in R. We discarded PCR replicates if they fell above the $95^{th}$ quantile of intra-sample dissimilarities for the sequencing run, or if they had a dissimilarity greater than the smallest between-sample dissimilarity. We retained only fecal samples with at least two PCR replicates remaining after this procedure, and we merged those replicates by averaging the number of reads per mOTU. Finally, to avoid false positives arising from low-level contamination or analytical error, we discarded mOTUs from the replicate-averaged sample if they accounted for > 1% of the sample's reads.

Mammal DNA sequences, used to verify samples' species identity, were curated with OBITools (v.1.2.11; Boyer et al. 2016) following the same procedures described above. The protocol differed in that the expected barcode length was set to 40–140 bp, all non-singleton sequences were retained, and mOTUs were assigned to taxa based only on a global reference database for mammals generated by *in silico* PCR and the European Molecular Biology Laboratory nucleotide sequence database (release 143). Secondary processing of these results involved assigning a species-of-origin to each sample. Following Pansu et al. (2022), we used five criteria for these assignments: (*i*) the sample had a read depth >350, (*ii*) the most common sequence accounted ≥50% of the sample's reads, (*iii*) the most common sequence matched a reference database sequence with ≥98% similarity (0 or 1 bp difference), (*iv*) only one Latin binomial was associated with the best match in the reference database, (*v*) the most common sequence was at least twice as abundant as the next most common sequence. Samples satisfying these criteria were assigned to the mammal species associated with the most abundant sequence. If a sample failed two or more of these criteria, we did not assign a species; data sets *11* and *12* include five samples where the mammal species of origin is 'Unknown'.

Tertiary processing involved merging data across sequencing runs, using mammal DNA results to update species identities, and integrating metadata. First, samples that were sequenced twice in separate batches were identified, and only the more recent duplicate was retained. We then merged data from different sequencing runs into one table and harmonized sample-naming conventions. Samples from other studies included in the same sequencing runs, or samples that were mislabeled during laboratory analyses, were removed and metadata from each year were harmonized and merged. For genetically verified samples, mammalian species of origin was added to the metadata, replacing any provisional field-based identification.

The penultimate processing step involved refining the taxonomic assignments of the most common plant mOTUs in the data set using additional repositories of DNA sequence data in conjunction with information on the geographic distribution of plant species. First, we compiled information on plants known to occur in PNG ('PNG flora') by combining occurrence records from PNG's Biodiversity Database (accessed 5 October 2023) and the Flora of Mozambique occurrence

database (https://www.mozambiqueflora.com/; accessed 18 October 2023). We harmonized the names of these records using the nomenclatural standards of Kew Botanical Garden's Plants of the World Online (POWO; https://powo.science.kew.org/; accessed 27 November 2023). We downloaded the list of species known to occur in Mozambique ('Mozambique flora') from POWO (accessed 10 November 2023). Second, we selected the most abundant mOTUs in the data set for deeper investigation. We first converted mOTU read counts per sample to relative read abundances (RRA) to standardize the range of mOTU abundance among samples. Our focal sequences were those that either (a) contributed to the top 90% of RRA across the entire data set; (b) contributed to the top 90% of any given species' diet; or (c) were among the 20 most abundant plants recorded in any given species' diet. Of 516 mOTUs, 145 satisfied one or more of these criteria. Third, we queried the 145 mOTUs using the *megaBLAST* tool of the National Center for Biotechnology Information (NBCI), allowing up to 5000 results. If this yielded no matches, we repeated the query using *blastn* (hereafter, we refer to both tools as *BLAST*). We downloaded all results with both 100% query cover and 100% percent match to the query sequence; if no results satisfied these criteria, the sequence with the minimum (i.e., best) E-value was downloaded (accessed 15–22 November 2023). These results were then harmonized with POWO nomenclature to facilitate matching to occurrence records from PNG and Mozambique. Fourth, we sorted the *BLAST* results according to whether taxa were known to occur in the PNG flora, Mozambique flora, or neither. Last, we compared the matching between each barcode and the local reference database to the matching against geographically refined *BLAST* results. For this, we first harmonized taxon names in the local DNA reference library ('local DB') using POWO conventions. We then refined the taxonomic identification based on the following decision rules:

- If no *BLAST* match, then use local DB assignment.
- If both *BLAST* and local DB return perfect matches and *BLAST* matches are in the PNG flora, then combine assignments.
- If both databases return perfect matches but only one has a species-level assignment, use the species-level assignment.
- If both return perfect matches but no *BLAST* matches are in the PNG flora, then use local DB assignment.
- If both return perfect matches but neither has species-level assignments from PNG, then use *BLAST* matches from species in the Mozambique flora.

- If only *BLAST* has a perfect match to a species recorded from PNG, then use that assignment.
- If only *BLAST* has a perfect match, but the species is not in the PNG flora, then use *BLAST* assignment to species present in Mozambique.
- If only *BLAST* has a perfect match, but none of the matches are recorded from PNG or Mozambique, then use local DB assignment.
- If only local DB has a perfect match, then use local DB assignment.
- If both databases have imperfect matches, but local DB has a higher percent match and a species-level identification, use local DB.
- If both have imperfect matches, and local DB has a higher percent match, but does not have a species-level identification, then search PNG plant list for the taxon and assign all taxa nested within that taxon.
- If both have imperfect matches, but *BLAST* has a higher percent identity to a species in the PNG flora, then use *BLAST* match from PNG.
- If both have imperfect matches, but *BLAST* has a higher percent identity to a species in Mozambique flora but not PNG flora, then use *BLAST* match from Mozambique.
- If both have imperfect matches, *BLAST* has a higher percent identity, but none of those taxa occur in Mozambique or PNG floras, then use *BLAST* matches but signal the issue with a leading asterisk.
- If neither database has an associated species-level identification, then search PNG flora for the taxon and assign all taxa nested within that taxon.

If more than two species were assigned to an mOTU following this procedure, then we assigned the lowest shared taxonomic rank including all species, as per NCBI's Taxonomy Browser. If a species was the sole assignment to one mOTU but was also part of a group of taxa assigned to another mOTU, then it was assigned to the mOTU for which it was the sole match and excluded from the final assignment for the other mOTU. The identity assigned to each mOTU included the final assignment (resulting from the above procedure for the top 145 mOTUs or the local DB match for the remainder), the botanical family name, and the DNA sequence belonging to each mOTU.

The last step of processing was to create a version of the data set where each sample comprised the same number of reads, to facilitate comparison among samples. We did this by randomly rarefying each sample down to the lowest number of reads in the data set ($n$ = 979). This threshold is lower than the 1000-read threshold imposed earlier in the bioinformatic pipeline owing to the exclusion of mOTUs accounting for < 1% of each sample's reads. We repeated this random process 1000 times and used the average resulting mOTU composition for each sample in data set *11*. These values were converted to RRA by dividing the rarefied abundance of each mOTU in each sample by the total number of rarefied reads per sample ($n$ = 979). An unrarefied version of the data set without conversion into RRA is deposited as data set *12*.

**E. Archiving:**

**1. Archival procedures:** Data are archived in several redundant locations (both publicly available and privately hosted) to guarantee long-term access to users worldwide.

**2. Redundant archival sites:** Data are stored in: this *Ecology* data paper, Data Dryad repository (DOI: 10.5061/dryad.m905qfv85), Movebank (GPS movement data; Study ID: 7359354918), and institutional long-term storage platforms. The original data from which these products are derived are stored on Princeton and the University of California Merced's high-performance computing clusters and the University of Idaho's OneDrive. Subsets of the data are also stored in Dryad repositories associated with previous publications:

https://doi.org/10.5061/dryad.h4r1003;
https://doi.org/10.5061/dryad.63tj806;
https://doi.org/10.5061/dryad.6v5v88g;
https://doi.org/10.5061/dryad.sxksn02zc;
https://doi.org/10.5061/dryad.qfttdz0h4;
https://doi.org/10.5061/dryad.4f4qrfjdk;
https://doi.org/10.5061/dryad.brv15dvcj;
https://doi.org/10.5061/dryad.9ghx3ffks;
https://doi.org/10.5061/dryad.crjdfn364;
https://doi.org/10.5061/dryad.w0vt4b936.

**F. Publications and results:**

Atkins, J.L., Long, R.A., Pansu, J., Daskin, J.H., Potter, A.B., Stalmans, M.E., Tarnita, C.E. and Pringle, R.M., 2019. Cascading impacts of large-carnivore extirpation in an African ecosystem. *Science*, *364*, pp.173-177.

Becker, J.A., Hutchinson, M.C., Potter, A.B., Park, S., Guyton, J.A., Abernathy, K., Americo, V.F., da Conceição, A., Kartzinel, T.R., Kuziel, L. Leonard, N.E., Lorenzi, E., Martins, N.C., Pansu, J., Scott, W.L., Stahl, M.K., Torrens, K.R., Stalmans, M.E., Long, R.A., Pringle, R.M., 2021. Ecological and behavioral mechanisms of density-dependent habitat expansion in a recovering African ungulate population. *Ecological Monographs*, *91*, e01476.

Buschke, F.T., Codron, D., Pringle, R.M., and Spaak, J. 2026. A theoretical framework for multispecies coexistence in large herbivores based on functional traits and dietary data. *Ecology Letters*, *in press*.

Branco, P.S., Merkle, J.A., Pringle, R.M., Pansu, J., Potter, A.B., Reynolds, A., Stalmans, M. and Long, R.A., 2019. Determinants of elephant foraging behaviour in a coupled human-natural system: Is brown the new green?. *Journal of Animal Ecology*, *88*, pp.780-792.

Branco, P.S., Merkle, J.A., Pringle, R.M., King, L., Tindall, T., Stalmans, M. and Long, R.A., 2020. An experimental test of community-based strategies for mitigating human–wildlife conflict around protected areas. *Conservation Letters*, *13*, e12679.

Daskin, J.H., Becker, J.A., Kartzinel, T.R., Potter, A.B., Walker, R.H., Eriksson, F.A., Buoncore, C., Getraer, A., Long, R.A. and Pringle, R.M., 2023. Allometry of behavior and niche differentiation among congeneric African antelopes. *Ecological Monographs*, *93*, e1549.

Guyton, J.A., Pansu, J., Hutchinson, M.C., Kartzinel, T.R., Potter, A.B., Coverdale, T.C., Daskin, J.H., da Conceição, A.G., Peel, M.J., Stalmans, M.E. and Pringle, R.M., 2020. Trophic rewilding revives biotic resistance to shrub invasion. *Nature Ecology & Evolution*, *4*, pp.712-724.

Hutchinson, M.C., Walker, R.H., Abraham, J.O., Becker, J.A., Gonçalves, D.D., Nutter, C.M., Pansu, J., Phillips, E.M., Potter, A.J.P., Tenente, B., Stalmans, M.E., Long, R.A., and Pringle, R.M. 2026. Ecology of community reassembly: Movements and diets of megafauna during a decade of restoration in Mozambique. *Ecology* (this article).

Pansu, J., Guyton, J.A., Potter, A.B., Atkins, J.L., Daskin, J.H., Wursten, B., Kartzinel, T.R. and Pringle, R.M., 2019. Trophic ecology of large herbivores in a reassembling African ecosystem. *Journal of Ecology*, *107*, pp.1355-1376.

Pansu, J., Hutchinson, M.C., Anderson, T.M., Te Beest, M., Begg, C.M., Begg, K.S., Bonin, A., Chama, L., Chamaillé-Jammes, S., Coissac, E., Cromsigt, J.P., Demmel, M.Y., Donaldson, J.E., Guyton, J.A., Hansen, C.B., Imakando, C.I., Iqbal, A., Kalima, D.F., Kerley, G.I.H., Kurukura, S., Landman, M., Long, R.A., Munuo, I.N., Nutter, C.M., Parr, C.L., Potter, A.B., Siachoono, S., Taberlet, P., Waiti, E., Kartzinel, T.R., and Pringle, R.M., 2022. The generality of cryptic dietary niche differences in diverse large-herbivore assemblages. *Proceedings of the National Academy of Sciences*, *119*, e2204400119.

Potter, A.B., Hutchinson, M.C., Pansu, J., Wursten, B., Long, R.A., Levine, J.M. and Pringle, R.M., 2022. Mechanisms of dietary resource partitioning in large-herbivore assemblages: A plant-trait-based approach. *Journal of Ecology*, *110*, pp.817-832.

Potter, A.B. and Pringle, R.M., 2023. Revisiting the Jarman–Bell Principle. In H.H.T. Prins and I.J. Gordon (eds) *The Equids: A Suite of Splendid Species* (pp. 171-207). Springer International Publishing.

Pringle, R.M. and Hutchinson, M.C., 2020. Resolving food-web structure. *Annual Review of Ecology, Evolution, and Systematics*, *51*, pp.55-80.

Tucker, M.A., and 169 co-authors. 2023. Behavioral responses of terrestrial mammals to COVID-19 lockdowns. *Science*, *380*, pp. 1059–1064.

Van Driessche, J.A., S. Chamaillé-Jammes, C.M. Nutter, A.B. Potter, R.M. Pringle, and R.A. Long. 2025. Water economics of savanna herbivores: how much does plant moisture matter? *Journal of Animal Ecology*, *94*, pp. 670-681.

Walker, R.H., Hutchinson, M.C., Potter, A.B., Becker, J.A., Long, R.A. and Pringle, R.M., 2023. Mechanisms of individual variation in large herbivore diets: Roles of spatial heterogeneity and state-dependent foraging. *Ecology*, *104*, e3921.

Walker, R.H., Hutchinson, M.C., Becker, J.A., Daskin, J.H., Gaynor, K.M., Palmer, M.S., Gonçalves, D.D., Stalmans, M.E., Denlinger, J., Bouley, P., Angela, M., Paulo, A., Potter, A.B., Arumoogum, N., Parrini, F., Marshal, J.P., Pringle, R.M., Long, R.A., 2023. Trait-based sensitivity of large mammals to a catastrophic tropical cyclone. *Nature*, *623*, pp.757-764.

**G. History of data set usage:** Various subsets of the data collated here have been used in the previously published work cited above. Some data are also currently in use by the authors of this data paper to address questions related to the dietary and movement behaviors of large mammalian herbivores and their community assembly processes.

**1. Data request history:** To our knowledge, this data set has not been used in studies except those authored or coauthored by at least one author listed on this contribution.

**2. Data set update history:** Subsets of the data collated here have been featured in publications, which are listed in *Class V.F*. In those publications, specific data filtering methods are reported, which vary in respects from those used here (e.g., rarefied read depth). For DNA metabarcoding data sets, the filtering procedures and DNA reference libraries have evolved since the original publications with these data, in part owing the ever-increasing breadth and depth of reference DNA sequence data, as described in *Class V.D.ii*. The procedures applied here unify the filtering protocol across all currently available samples and their associated data and identify all sequences with reference to the same DNA reference libraries to facilitate future comparisons.

**3. Review history:** Data were last reviewed and entered by M.C.H. and R.H.W. in November 2025 while revising this contribution in accordance with reviewer feedback and were subsequently reviewed and several minor errors corrected by M.C.H., R.H.W., R.A.L. and R.M.P. in February 2026.

**4. Questions and comments from secondary users:** NA

## References

Anderson, J. L. 1986. Age determination of the nyala *Tragelaphus angasi*. South African Journal of Wildlife Research 16:82–90.

Anderson, T.M., S. A. Hepler, R. M. Holdo, J. E. Donaldson, R. J. Erhardt, G. C. Hopcraft, M. C. Hutchinson, S. E. Huebner, T. A. Morrison, J. Muday, I. N. Munuo, M. S. Palmer, J. Pansu, R. M. Pringle, R. Sketch, C. Packer. 2024. Interplay of competition and facilitation in grazing succession by migrant Serengeti herbivores. Science 383:728–788.

Atkins, J. L., R. A. Long, J. Pansu, J. H. Daskin, A. B. Potter, M. E. Stalmans, C. E. Tarnita, and R. M. Pringle. 2019. Cascading impacts of large-carnivore extirpation in an African ecosystem. Science 364:173–177.

Bagchi, S., and M. E. Ritchie. 2010. Body size and species coexistence in consumer–resource interactions: A comparison of two alternative theoretical frameworks. Theoretical Ecology 5:141–151.

Becker, J. A., M. C. Hutchinson, A. B. Potter, S. Park, J. A. Guyton, K. Abernathy, V. F. Americo, A. da Conceição, T. R. Kartzinel, L. Kuziel, N. E. Leonard, E. Lorenzi, N. C. Martins, J. Pansu, W. L. Scott, M. K. Stahl, K. R. Torrens, M. E. Stalmans, R. A. Long, and R. M. Pringle. 2021. Ecological and behavioral mechanisms of density-dependent habitat expansion in a recovering African ungulate population. Ecological Monographs 91:e01476.

Bouley, P., A. Paulo, M. Angela, C. Du Plessis, and D. G. Marneweck. 2021. The successful reintroduction of African wild dogs (*Lycaon pictus*) to Gorongosa National Park, Mozambique. PLOS ONE 16:e0249860.

Bouley, P., M. Poulos, R. Branco, N. H. Carter. 2018. Post-war recovery of the African lion in response to large-scale ecosystem restoration. Biological Conservation 227:233–242.

Boyer, F., C. Mercier, A. Bonin, Y. Le Bras, P. Taberlet, and E. Coissac. 2016. obitools: a unix-inspired software package for DNA metabarcoding. Molecular Ecology Resources 16:176–182.

Branco, P. S., J. A. Merkle, R. M. Pringle, L. King, T. Tindall, M. Stalmans, and R. A. Long. 2020. An experimental test of community-based strategies for mitigating human–wildlife conflict around protected areas. Conservation Letters 13:e12679.

Branco, P. S., J. A. Merkle, R. M. Pringle, J. Pansu, A. B. Potter, A. Reynolds, M. Stalmans, and R. A. Long. 2019. Determinants of elephant foraging behaviour in a coupled human-natural

system: Is brown the new green? Journal of Animal Ecology 88:780–792.

Buschke, F. T., D. Codron, R. M. Pringle, J. Spaak. 2026. A theoretical framework for multispecies coexistence in large herbivores based on functional traits and dietary data. Ecology Letters (in press).

Campbell-Staton, S. C., B. J. Arnold, D. Gonçalves, P. Granli, J. Poole, R. A. Long, and R. M. Pringle. 2021. Ivory poaching and the rapid evolution of tusklessness in African elephants. Science 374:483–487.

Daskin, J. H., J. A. Becker, T. R. Kartzinel, A. B. Potter, R. H. Walker, F. A. A. Eriksson, C. Buoncore, A. Getraer, R. A. Long, and R. M. Pringle. 2023. Allometry of behavior and niche differentiation among congeneric African antelopes. Ecological Monographs 93:e1549.

Daskin, J. H., and R. M. Pringle. 2018. Warfare and wildlife declines in Africa's protected areas. Nature 553:328–332.

Daskin, J. H., M. Stalmans, and R. M. Pringle. 2016. Ecological legacies of civil war: 35-year increase in savanna tree cover following wholesale large-mammal declines. Journal of Ecology 104:79–89.

Deng, J., O. X. Cordero, T. Fukami, S. A. Levin, R. M. Pringle, R. Solé, and S. Saavedra. 2024. The development of ecological systems along paths of least resistance. Current biology : CB 34:4813–4823.e14.

Furstenburg, D. 2010. Focus on Bushbuck Tragelaphus scriptus. GeoWild Consult (Pty) Ltd.

Furstenburg, D. 2016. Focus on Greater Kudu Tragelaphus strepsiceros. GeoWild Consult (Pty) Ltd.

Giguet-Covex, C., J. Pansu, F. Arnaud, P.-J. Rey, C. Griggo, L. Gielly, I. Domaizon, E. Coissac, F. David, P. Choler, J. Poulenard, and P. Taberlet. 2014. Long livestock farming history and human landscape shaping revealed by lake sediment DNA. Nature Communications 5:1–7.

Gonçalves, D. 2023. Living with Giants: Implications of Elephant Habitat Choice on Human-Elephant Conflict and Coexistence. Ph.D. dissertation, University of Kent, Kent, UK.

Guyton, J. A., J. Pansu, M. C. Hutchinson, T. R. Kartzinel, A. B. Potter, T. C. Coverdale, J. H. Daskin, A. G. da Conceição, M. J. S. Peel, M. E. Stalmans, and R. M. Pringle. 2020. Trophic rewilding revives biotic resistance to shrub invasion. Nature Ecology & Evolution 4:712–724.

Hassanin, A., M. L. Houck, T. D., B. Kadjo, H. Davis, and R. A. 2018. Multi-locus phylogeny of

the tribe Tragelaphini (Mammalia, Bovidae) and species delimitation in bushbuck: Evidence for chromosomal speciation mediated by interspecific hybridization. Molecular Phylogenetics and Evolution 129:96–105.

Hatton, I. A., K. S. McCann, J. M. Fryxell, T. J. Davies, M. Smerlak, A. R. E. Sinclair, and M. Loreau. 2015. The predator-prey power law: Biomass scaling across terrestrial and aquatic biomes.

Kartzinel, T. R., P. A. Chen, T. C. Coverdale, D. L. Erickson, W. J. Kress, M. L. Kuzmina, D. I. Rubenstein, W. Wang, and R. M. Pringle. 2015. DNA metabarcoding illuminates dietary niche partitioning by African large herbivores. Proceedings of the National Academy of Sciences 112:8019–8024.

Lötter, M., J. Burrows, K. Jones, E. Duarte, H. Costa, W. McCleland, M. Stalmans, E. Schmidt, I. Darbyshire, S. Richards, M. Soares, H. Grantham, H. Matimele, C. Sousa, T. Alves, R. Zolho, D. Nicolau, N. Ribeiro, C. Macamo, A. Massingue, and S. Bandeira. 2023. Historical vegetation map and red list of ecosystems assessment for Mozambique – Final report. USAID / SPEED+, AFD/FFEM. Maputo, Mozambique.

Millar, J. S., and G. J. Hickling. 1990. Fasting Endurance and the Evolution of Mammalian Body Size. Functional Ecology 4:5–12.

Morley, R., and I. Convery. 2014. Restoring Gorongosa: Some Personal Reflections. *in* Convery, I., Corsane, G., and P. Davis, editors. Displaced Heritage: Responses to Disaster, Trauma, and Loss. Boydell Press, Woodbridge.

Oksanen, J., F. G. Blanchet, R. Kindt, P. Legendre, P. R. Minchin, R. B. O'Hara, G. L. Simpson, P. Solymos, M. H. H. Stevens, H. Wagner, and M. J. Oksanen. 2013. Package "vegan": Community ecology package.

Pansu, J., J. A. Guyton, A. B. Potter, J. L. Atkins, J. H. Daskin, B. Wursten, T. R. Kartzinel, and R. M. Pringle. 2019. Trophic ecology of large herbivores in a reassembling African ecosystem. Journal of Ecology 107:1355–1376.

Pansu, J., M. C. Hutchinson, T. M. Anderson, M. te Beest, C. M. Begg, K. S. Begg, A. Bonin, L. Chama, S. Chamaillé-Jammes, E. Coissac, J. P. G. M. Cromsigt, M. Y. Demmel, J. E. Donaldson, J. A. Guyton, C. B. Hansen, C. I. Imakando, A. Iqbal, D. F. Kalima, G. I. H. Kerley, S. Kurukura, M. Landman, R. A. Long, I. N. Munuo, C. M. Nutter, C. L. Parr, A. B. Potter, S. Siachoono, P. Taberlet, E. Waiti, T. R. Kartzinel, and R. M. Pringle. 2022. The

generality of cryptic dietary niche differences in diverse large-herbivore assemblages. Proceedings of the National Academy of Sciences 119:e2204400119.

Park, S., K. H. Aschenbach, M. Ahmed, W. L. Scott, N. E. Leonard, K. Abernathy, G. Marshall, M. Shepard, and N. C. Martins. 2019. Animal-borne wireless network: Remote imaging of community ecology. Journal of Field Robotics 36:1141–1165.

Phillips, E. M. 2026. Underdogs: Ecology and intraguild dynamics of African mesocarnivores. Ph.D. dissertation, Princeton University, Princeton, New Jersey, USA.

Potter, A. B., M. C. Hutchinson, J. Pansu, B. Wursten, R. A. Long, J. M. Levine, and R. M. Pringle. 2022. Mechanisms of dietary resource partitioning in large-herbivore assemblages: A plant-trait-based approach. Journal of Ecology 110:817–832.

Prins, H. H. T. 2016. Interspecific resource competition in antelopes: search for evidence. Pp. 51–77 *in* J. Bro-Jørgensen and D. P. Mallon, editors. Antelope conservation: from diagnosis to action. Wiley-Blackwell, London.

Pringle, R. M. 2017. Upgrading protected areas to conserve wild biodiversity. Nature 546:91–99.

Pringle, R. M., and D. Gonçalves. 2022. Rewilding Case Study: Gorongosa National Park, Mozambique. Pp. 180–190 *in* S. Hawkins, I. Convery, S. Carver, and R. Beyers, editors. Routledge Handbook of Rewilding. Routledge, London.

Sikes, R. S., and Animal Care and Use Committee of the American Society of Mammalogists. 2016. 2016 Guidelines of the American Society of Mammalogists for the use of wild mammals in research and education. Journal of Mammalogy 97:1013.

Silva, V., 2020, Exploring Cooperative Behaviors Among the Sena of Gorongosa National Park, Mozambique, and the Dolgan/Nganasan of Ust'-Avam, Siberia. MA thesis, Boise State University, Boise, ID, United States of America.

Simpson, C. D. 1966. Tooth eruption, growth and ageing criteria in greater kudu *Tragelaphus strepsiceros*, Pallas. Arnotdia 2:1–12.

Simpson, C. D. 1971. Horn growth as a potential age criterion in some southern African antelopes. South African Journal of Wildlife Research 1:20–25.

Simpson, C. D. 1972. Some characteristics of Tragelaphine horn growth and their relationships to age in greater kudu and bushbuck. Journal of Southern African Wildlife Management Association 2:1–8.

Sinclair, A. R. E., S. Mduma, and J. S. Brashares. 2003. Patterns of predation in a diverse predator–

prey system. Nature 425:288–290.

Stalmans, M., and R. Beilfuss. 2008. Landscapes of the Gorongosa National Park. Gorongosa National Park.

Stalmans, M. E., T. J. Massad, M. J. S. Peel, C. E. Tarnita, and R. M. Pringle. 2019. War-induced collapse and asymmetric recovery of large-mammal populations in Gorongosa National Park, Mozambique. PLOS ONE 14:e0212864.

Stalmans, M., M. Peel, and D. Gonçalves. 2022. Aerial Wildlife Count of the Gorongosa National Park. Gorongosa National Park.

Stalmans, M., and M. Peel. 2024. Aerial Wildlife Count of the Gorongosa National Park. Gorongosa National Park.

Staver, A. C., S. Archibald, and S. A. Levin. 2011. The global extent and determinants of savanna and forest as alternative biome states. Science 334:230–232.

Stoler, N., and A. Nekrutenko. 2021. Sequencing error profiles of Illumina sequencing instruments. NAR Genomics and Bioinformatics 3.

Taberlet P., E. Coissac, F. Pompanon, L. Gielly, C. Miquel, A. Valentini, T. Vermat, G. Corthier, C. Brochmann, E. Willerslev. 2007. Power and limitations of the chloroplast *trn*L (UAA) intron for plant DNA barcoding. Nucleic Acids Research. 35:e14.

Taberlet, P., A. Bonin, L. Zinger, and E. Coissac. 2018. Environmental DNA: For Biodiversity Research and Monitoring. Oxford University Press, Oxford, England.

Tarnita, C. E., J. A. Bonachela, E. Sheffer, J. A. Guyton, T. C. Coverdale, R. A. Long, and R. M. Pringle. 2017. A theoretical foundation for multi-scale regular vegetation patterns. Nature 541:398–401.

Tinley, K. L. 1977. Montane to Mangrove: Framework of the Gorongosa Ecosystem. Ph.D. dissertation, University of Pretoria, Pretoria, South Africa.

Tucker, M.A., and 169 co-authors. 2023. Behavioral responses of terrestrial mammals to COVID-19 lockdowns. Science 380:1059–1064.

Van Driessche, J.A., S. Chamaillé-Jammes, C.M. Nutter, A.B. Potter, R.M. Pringle, and R.A. Long. 2025. Water economics of savanna herbivores: how much does plant moisture matter? Journal of Animal Ecology 94:670-681.

Walker, R. H., M. C. Hutchinson, J. A. Becker, J. H. Daskin, K. M. Gaynor, M. S. Palmer, D. D. Gonçalves, M. E. Stalmans, J. Denlinger, P. Bouley, M. Angela, A. Paulo, A. B. Potter, N.

Arumoogum, F. Parrini, J. P. Marshal, R. M. Pringle, and R. A. Long. 2023a. Trait-based sensitivity of large mammals to a catastrophic tropical cyclone. Nature 623:757–764.

Walker, R. H., M. C. Hutchinson, A. B. Potter, J. A. Becker, R. A. Long, and R. M. Pringle. 2023b. Mechanisms of individual variation in large herbivore diets: Roles of spatial heterogeneity and state-dependent foraging. Ecology 104:e3921.
